# Identification of cancer mini-drivers by deciphering selective landscape in the cancer genome

**DOI:** 10.1093/bib/bbaf694

**Published:** 2026-01-09

**Authors:** Xunuo Zhu, Wenyi Zhao, Siqi Wang, Jingwen Yang, Jingqi Zhou, Binbin Zhou, Ji Cao, Bo Yang, Zhan Zhou, Xun Gu

**Affiliations:** State Key Laboratory of Advanced Drug Delivery and Release Systems & Zhejiang Provincial Key Laboratory of Anti-Cancer Drug Research, College of Pharmaceutical Sciences, Zhejiang University, 866 Yuhangtang Rd, Xihu District, Hangzhou, Zhejiang 310058, P.R. China; State Key Laboratory of Advanced Drug Delivery and Release Systems & Zhejiang Provincial Key Laboratory of Anti-Cancer Drug Research, College of Pharmaceutical Sciences, Zhejiang University, 866 Yuhangtang Rd, Xihu District, Hangzhou, Zhejiang 310058, P.R. China; Innovation Institute for Artificial Intelligence in Medicine & MOE Engineering Research Center of Innovative Anticancer Drugs, Zhejiang University, 291 Fucheng Rd, Qiantang District, Hangzhou, Zhejiang 310018, P.R. China; State Key Laboratory of Advanced Drug Delivery and Release Systems & Zhejiang Provincial Key Laboratory of Anti-Cancer Drug Research, College of Pharmaceutical Sciences, Zhejiang University, 866 Yuhangtang Rd, Xihu District, Hangzhou, Zhejiang 310058, P.R. China; School of Medicine, Indiana University, 340 West 10th Street, Indianapolis, IN 46202, USA; School of Public Health, Shanghai Jiao Tong University School of Medicine, 227 South Chongqing Rd, Huangpu District, Shanghai 200025, P.R. China; School of Computer and Computing Science, Hangzhou City University, 51 Huzhou St, Gongshu District, Hangzhou, Zhejiang 310015, P.R. China; State Key Laboratory of Advanced Drug Delivery and Release Systems & Zhejiang Provincial Key Laboratory of Anti-Cancer Drug Research, College of Pharmaceutical Sciences, Zhejiang University, 866 Yuhangtang Rd, Xihu District, Hangzhou, Zhejiang 310058, P.R. China; Innovation Institute for Artificial Intelligence in Medicine & MOE Engineering Research Center of Innovative Anticancer Drugs, Zhejiang University, 291 Fucheng Rd, Qiantang District, Hangzhou, Zhejiang 310018, P.R. China; State Key Laboratory of Advanced Drug Delivery and Release Systems & Zhejiang Provincial Key Laboratory of Anti-Cancer Drug Research, College of Pharmaceutical Sciences, Zhejiang University, 866 Yuhangtang Rd, Xihu District, Hangzhou, Zhejiang 310058, P.R. China; Innovation Institute for Artificial Intelligence in Medicine & MOE Engineering Research Center of Innovative Anticancer Drugs, Zhejiang University, 291 Fucheng Rd, Qiantang District, Hangzhou, Zhejiang 310018, P.R. China; School of Medicine, Hangzhou City University, 51 Huzhou St, Gongshu District, Hangzhou, Zhejiang 310015, P.R. China; State Key Laboratory of Advanced Drug Delivery and Release Systems & Zhejiang Provincial Key Laboratory of Anti-Cancer Drug Research, College of Pharmaceutical Sciences, Zhejiang University, 866 Yuhangtang Rd, Xihu District, Hangzhou, Zhejiang 310058, P.R. China; Innovation Institute for Artificial Intelligence in Medicine & MOE Engineering Research Center of Innovative Anticancer Drugs, Zhejiang University, 291 Fucheng Rd, Qiantang District, Hangzhou, Zhejiang 310018, P.R. China; The Fourth Affiliated Hospital, Zhejiang University School of Medicine, N1 Shangcheng Rd, Yiwu, Zhejiang 322000, P.R. China; Department of Genetics, Development and Cell Biology, Iowa State University, 2437 Pammel Drive, Ames, IA 50011, USA

**Keywords:** cancer evolution, somatic mutation, mini-driver genes, selection pressure, *C_N_/C_S_*-calculator

## Abstract

Cancer development is driven by somatic evolution and clonal selection. However, traditional selective pressure analysis methods have treated all sites within a gene equally, such a gene-level model oversimplifies the complexity of cancer evolution. In this study, we introduced *C_N_/C_S_*-calculator, a novel site-specific method that can capture selective pressures acting across different gene sites. By deciphering the interplay between the selection pattern and the function of a gene in oncogenesis, *C_N_/C_S_*-calculator uncovers a unique class of mini-driver genes, which exhibit weak positive selection, with certain critical sites providing context-dependent promoter effects on the fitness of cancer subclones while others are constrained by evolutionary conservation. Our method emphasizes the importance of site-specific analysis in uncovering how subtle evolutionary forces shape cancer biology. The refined understanding offers new insights into the mechanisms of cancer heterogeneity and molecular evolution, with potential implications for advancing therapeutic strategies and prognostic assessments.

## Introduction

The concept that cancer development is essentially an evolutionary process has been widely accepted [1, 2]. Based on the theory of molecular evolution, many studies have conducted detailed analyses of selection patterns during cancer evolution [3–13] and identified genes that are closely related to cancer development by calculating evolutionary selection pressures [5, 10, 14]. For a long time, researchers have focused on positively selected genes, which are more likely to provide a growth advantage to tumor cells and are classified as cancer driver genes [5, 15]. Most of the remaining genes are considered to escape from negative selection and are thus irrelevant passenger genes [16–18].

However, such a dichotomous model is insufficient to explain the complexity of cancer [19]. It has been found that inter-tumor heterogeneity is much higher than previously thought, with only a small fraction of genes recurrently mutated, and the lack of driver genes in many tumor samples suggests that some molecular mechanisms underlying cancer progression are still buried in numerous passenger genes, waiting to be discovered. Intuitively, there is a set of mini-driver genes that lie in an ambiguous zone between positive and negative selection [20–24].

The polygenic mini-driver model suggests that, in contrast to the leapfrog evolution caused by driver mutations, mini-driver mutations can only provide a slow and gradual boost to the fitness of cancer subclones, which also provides a plausible explanation for the high degree of intratumoral heterogeneity [25]. By exploring the pathogenic mechanism of somatic mutations, many studies have coincidentally corroborated the concept of mini-drivers, which may provide conditional, synergistic, fine-tuned or additive functional alterations to driver genes [20–24, 26–29]. As examples of mini drivers exerting conditional or synergistic effects, both Auslander *et al.* [20] and Yavuz *et al.* [28] identified interactors of the major cancer drivers, whose oncogenic activity is conditional to and associated with the occurrence of mutations in major drivers. Another type of mini-drivers may fine-tune an existing functional defect for insurance against catastrophic changes in environmental conditions, such as those induced by therapy [30]. For instances, menin inhibition exerts sufficient selection pressure in patients to drive the evolution of acquired resistance mutants in *MEN1* [27] and *ARAF* mutations confer resistance to the RAF inhibitor in melanoma [29]. As a final example for additive functional alterations of mini-drivers, Kumar *et al.* [19] showed that the aggregated effect of putative passengers, including undetected weak drivers, provides significant additional power for predicting cancerous phenotypes.

While all of these studies have proven the existence of mini-drivers, they have yet to define the basic principles for systematically identifying mini-driver genes. To address this gap, we first propose that mini-drivers occupy a unique position in the spectrum of selection pressures and typically possess three major features: (i) subject to weak positive selection, (ii) have at least one driver site contributing to oncogenic processes, and (iii) the remaining sites are under negative selection to maintain essential functions. Genes with features (i) and (ii) are defined as mini-driver genes, and mini-drivers that additionally match feature (iii) are further defined as conserved mini-driver genes.

Currently, developing comprehensive and accurate algorithms to predict mini-driver genes remains a great challenge due to the low accuracy and sensitivity of driver gene prediction algorithms as well as the inadequate understanding of the functional impact of mutations. Conventional selection pressure-based methods for driver gene prediction typically treat all sites of a gene equally, which may obscure intricate selection situations. For instance, within a gene, some sites may experience positive selection, indicating an adaptive change, whereas others may be under negative selection to preserve functional constraints. As a result, the overall selective pressure of genes tends to hover around one, reflecting a state of neutral selection [31]. Hence, there is an urgent need to propose new methods to compute the various selective pressures acting on different sites within genes and to comprehensively characterize the complex selective landscape in the cancer genome, which may contribute to the identification of mini-driver genes.

Here, to systematically identify mini-driver genes, we introduce the *C_N_/C_S_*-calculator, a two-component selective pressure analysis method designed to distinguish the selection pressure of different site components. The rationale behind *C_N_/C_S_*-calculator lies in the recognition that not all variants within a gene contribute equally to oncogenic processes, with some variants driving cancer progression while others are constrained by functional requirements. By comprehensively analyzing the selection landscape of the cancer genome, we uncovered not only classical positively selected driver genes, but also weakly positively selected mini-drivers. This refined understanding offers new insights into cancer molecular evolution and holds promise for advancing therapeutic strategies, prognostic assessments, and reshaping our perspectives on cancer etiology.

## Results

### The framework of selection pressure interpretation with *C_N_/C_S_*-calculator

**Figure 1:**
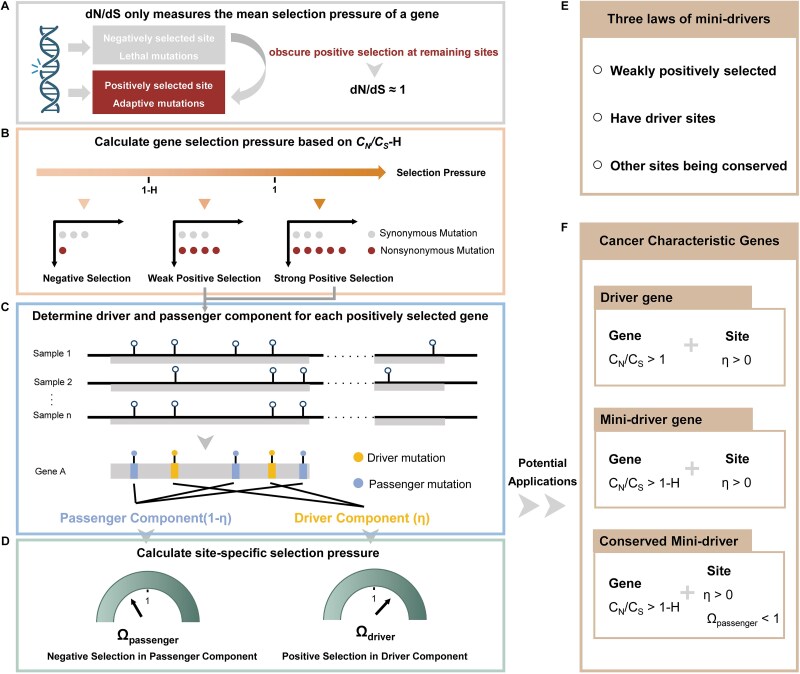
Schematic overview of the C_N_/C_S_-calculator. (A) The shortcoming of traditional selective pressure analysis methods, which treat all sites within a gene equally, simplifies the complexity of cancer evolution. (B–D) Analyzing the selection patterns of cancer genomes using C_N_/C_S_-calculator comprises three steps: (B) calculating gene selection pressure using C_N_/C_S_-H metrics, (C) determining driver and passenger component for positively and weak positively selected genes using two-component mixture model, (D) calculating selection pressure for driver component and passenger component respectively. (E–F) Together, these measures provide a comprehensive analytical framework for selective landscape, making it suitable to identify cancer characteristic genes in detail. (E) Three major selection pressure features of mini-driver genes. (F) Criteria for identifying cancer characteristic genes.

One of the most widely used indicator for the positive selection of an encoding gene in carcinogenesis is *C_N_/C_S_*, the ratio of the nonsynonymous mutation rate (*C_N_*) to the synonymous mutation rate (*C_S_*) in cancer samples [5, 7, 10, 32]. However, the estimated *C_N_/C_S_* ratio only measures the mean selection pressure across all sites in a gene, resulting in weak positive selection at individual sites being masked by those constrained by evolutionary conservation (Fig. 1A).

Therefore, instead of using *C_N_/C_S_* = 1 as an indicator of neutral selection, we developed *C_N_/C_S_*-calculator model, which consists of three modules to characterize the selection patterns of cancer genomes (Figs 1B–D). In the first modules of *C_N_/C_S_*-calculator, we introduced a new indicator *C_N_/C_S_*-H. Intuitively, *H* is defined as a relative measure of the variation in evolutionary rates among sites, reflecting how unevenly different sites in a gene are constrained or variable during evolution. Ranging from 0 to 1, a high value of *H* indicates a high degree of rate variation among sites, and vice versa [31, 33]. *C_N_/C_S_* values between 1-H and 1 indicate the detectable signal of positive selection in a gene with strong functional constraints (A detailed explanation is provided in the *Supplementary Methods Distinguishing different selection modes in cancer evolution using C_N_/C_S_-H*), while *C_N_/C_S_* values larger than one indicate dominant positive selection. These positively selected genes with *C_N_/C_S_* > 1-H, proceeded to the second module, a two-component mixture model that can calculate the proportion of driver components ($\eta$) and passenger components ($1-\eta$) in a gene. By combining the site components into the calculation of selective pressure, the third module attempts to portray the evolutionary dynamics of different types of gene sites (*Ω_driver_* for the selection pressure of driver component and *Ω_passenger_* for passenger component, denoted as *Ω_dri_* and *Ω_pass_*, respectively, in the following sections.). Through a detailed analysis of selection modes, the *C_N_/C_S_*-calculator seeks to elucidate the differential selective forces acting upon various sites within a gene, thereby shedding light on their functional significance in carcinogenesis, especially for those mini-driver genes (Fig. 1E and F).

### Landscape of selective pressure in the cancer genome

**Figure 2:**
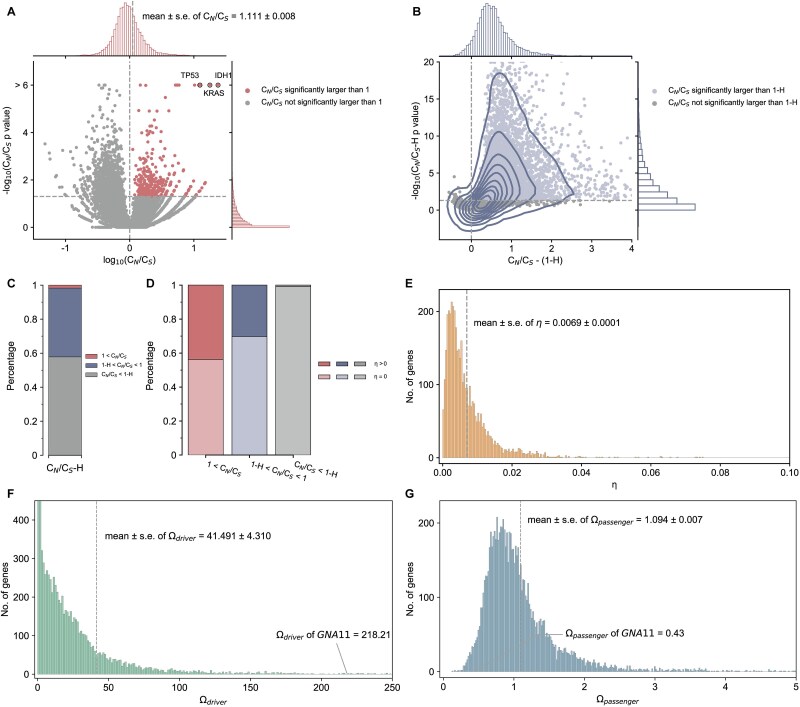
Distribution of selection pressure of genes in the cancer genome. (A) Distribution of C_N_/C_S_ values. The scatter shows the relationship between log_10_(C_N_/C_S_) values and corresponding -log_10_(C_N_/C_S_ p values). (B) The relationship between C_N_/C_S_ - (1-H) values and corresponding -log_10_(C_N_/C_S_-H *P*-values). C_N_/C_S_ - (1-H) greater than 0 corresponds to C_N_/C_S_ > 1-H. (C) The percentage of positively selected genes (C_N_/C_S_ > 1), weakly positively selected genes (1-H < C_N_/C_S_ < 1) and negatively selected genes (C_N_/C_S_ < 1 H) identified at the gene level. (D) Distribution of η values. (E) The proportion of genes that contain at least one driver site across different selection modes. (F) Distribution of Ω_driver_ values. (G) Distribution of Ω_passenger_ values.

According to the somatic mutation data extracted from the TCGA MC3 project [34], a total of 19,266 genes had at least one missense mutation, allowing further calculation of *C_N_/C_S_* values. The overall *C_N_/C_S_* value of these genes was close to, but slightly greater than one (mean ± s. e. = 1.111 ± 0.008) (Fig. 2A), which indicated that the cancer genome as a whole presents a picture of slight positive selection. Among these 19 266 genes, 333 had *C_N_/C_S_* values much greater than one (${\chi}^2$ test, *P* < .05), of which 29.1% were known driver genes (e.g. 12.44 for *TP53*, 17.76 for *KRAS* and 23.80 for *IDH1*), showing that cancer-associated genes were notably enriched in genes subjected to positive selection (*hypergeometric test*, *P* = 3.99 × 10^−26^). Of the remaining genes with *C_N_/C_S_* less than one, 7793 of them had *C_N_/C_S_* values significantly larger than 1-H (Fig. 2B), suggesting that ~40% of genes contain mutations that confer a selective advantage to cancer cells, but this advantage is masked by negative selection at conserved sites (Fig. 2C).

The proportion of genes with driver components (which means $\eta$ > 0) varied considerably among gene sets with different selection patterns, ranging from 36.7% in genes with *C_N_/C_S_* > 1 to 2.3% in genes with *C_N_/C_S_* < 1-H (Fig. 2D). Meanwhile, the estimated proportion of cancer-driving sites in a gene ($\eta$) was very small (mean ± s. e. = 0.0069 ± 0.0001) (Fig. 2E).

When taking site components into consideration, the selective pressure distributions of the driver and passenger components present a very different landscape. For genes with driver component, the mean *Ω_dri_* value was up to 41.491 ± 4.310 (mean ± s. e.), suggesting that strong positive selection is the dominant force shaping the evolutionary pattern of the driver component (Fig. 2F). But the mean *Ω_pass_* value was 1.094 ± 0.007 (mean ± s. e.), indicating that negative and neutral selection are the dominant forces shaping the evolutionary pattern of the passenger component (Fig. 2G). For example, the *Ω_pass_* value of *GNA11* is merely 0.43, reflecting its highly conserved nature as the α subunit for a G protein, but its *Ω_dri_* is as high as 218.21, consistent with the finding that an oncogenic mutation in it constitutively activate downstream signaling pathways [35, 36]. In conclusion, the *C_N_/C_S_*-calculator can simultaneously consider mutation frequency and mutation bias towards missense mutations to capture site-level positive selection. It also demonstrates enhanced sensitivity in detecting negative selection signals, making it a promising tool for identifying mini-drivers.

### Integration of selection modes in defining drivers and mini-drivers with different evolutionary origins

The three modules of *C_N_/C_S_*-calculator are exactly suitable for uncovering the three main features of mini-driver genes. The first module detects whether the gene is under positive selection, regardless of the presence of conserved sites; the second module detects whether the gene has driver component and the third module checks whether the rest of the sites are subject to functional constraints (Figs 1B–D). Therefore, we applied *C_N_/C_S_*-calculator on TCGA PanCanAtlas MC3 project to identify cancer characteristic genes under different selection modes. Specifically, the *C_N_/C_S_*-H module and the two-component mixture module jointly identified 129 positively selected genes (Supplementary Table S1) and 2354 weakly positively selected genes (Supplementary Table S2), both with driver components, which aligned with the concepts of “driver genes” and “mini-driver genes,” respectively. Among the 2354 mini-driver genes, the third module further identified 124 genes whose passenger component are subject to significantly functional constraints (Supplementary Table S3). In this study, we defined this collection of genes that rigorously fit the three main features of mini-drivers as “conserved mini-driver genes”. Although all these genes are nominally under positive selection, the distribution of *Ω_dri_* shows that the driver components of the driver genes have greater positive selection pressure (Supplementary Fig. S1) than mini-driver genes. The disruption of genes and processes that appeared in early metazoan (EM) life to enhance intercellular cooperation is expected to be a recurrent driver of carcinogenesis, as implicated by the widespread occurrence of cancer across the tree of multicellular life.

**Figure 3:**
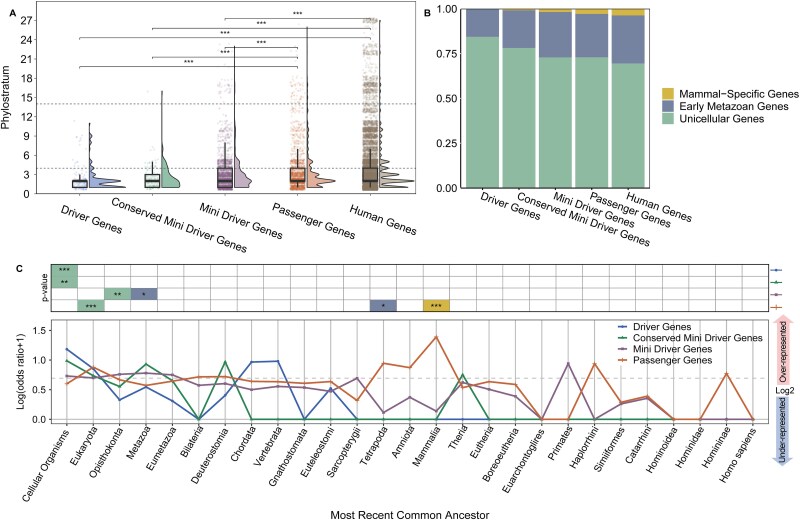
Phylostratigraphic analysis of cancer characteristic genes. (A) Phylostratigraphic tracking of five gene lists (driver genes, conserved mini driver genes, mini driver genes, passenger genes and all genes in human genome). Statistical significance was tested by Welch t-test. (B) The proportion of genes that originated from the UC, EM, MM phylostratum in each of the five gene lists. (C) Enrichment analysis of five gene lists on the phylostratigraphic map. Setting the “human genes” as baseline, phylostratigraphic representation of log-odds statistics of five gene lists are shown. The log(odds ratio + 1) of a phylostratum larger than log2 means that this phylostratum is over-represented in the gene list, and less than log2 means under-represented. The table at the top shows the significance of gene enrichment in each phylostratum for that gene list. The fill color green, blue and yellow represents UC, EM, MM phylostratum, respectively. Statistical significance was tested by hypergeometric distribution test (^*^P < .05; ^**^P < .01; ^***^P < .001).

Cancer has been suggested to result from an atavistic process, where primitive, highly conserved genes are reactivated to form molecular phenotypes similar to those of unicellular organisms [37–39]. Previous studies also have shown that evolutionarily older genes tend to evolve more slowly due to functional constraints, and are thus more likely to be associated with human diseases when mutated [37, 40–43]. Therefore, a deeper understanding of the evolutionary context of driver and mini-driver genes contributing to malignant transformation will be crucial for uncovering the molecular basis of tumor phenotypes. Here, we used phylostratr [44], a phylostratigraphy framework to infer evolutionary origin of driver and mini-drivers. Within the phylostratigraphy framework, phylostratum values serve as temporal indicators of gene origin, where lower values correspond to more ancient evolutionary ages [45]. After mapping all genes to their evolutionary origin, we found that the average phylostratigraphic ages for driver genes, conserved mini-driver genes, mini-driver genes, passenger genes (see *Methods*), and all genes in the human genome were 2.33, 2.47, 3.18, 3.52, and 3.76, respectively (Fig. 3A). Our results reveal that the study identified driver and mini-drivers exhibit a significantly more ancient age distribution than passenger genes and human genes (Fig. 3A and B) (*t-test*, *P* < .001 for all paired gene sets), implying a more fundamental role in the evolution of life. Notably, genes with evolutionary origins in cellular organisms were over-represented among the driver genes and conserved mini-driver genes, while the passenger genes displayed a younger age distribution, with the mammal-origin genes being over-represented among them (Fig. 3C). These results underscore the older evolutionary context of the identified driver and mini-drivers, implying that functional perturbations of these genes would promote the loss of multicellular features and the transition to a more “selfish” unicellular mode of life, leading to an increasingly atavistic malignant phenotype with increased selective advantage.

### *C_N_/C_S_*-calculator identified highly plausible cancer driver genes with potential clinical application

**Figure 4:**
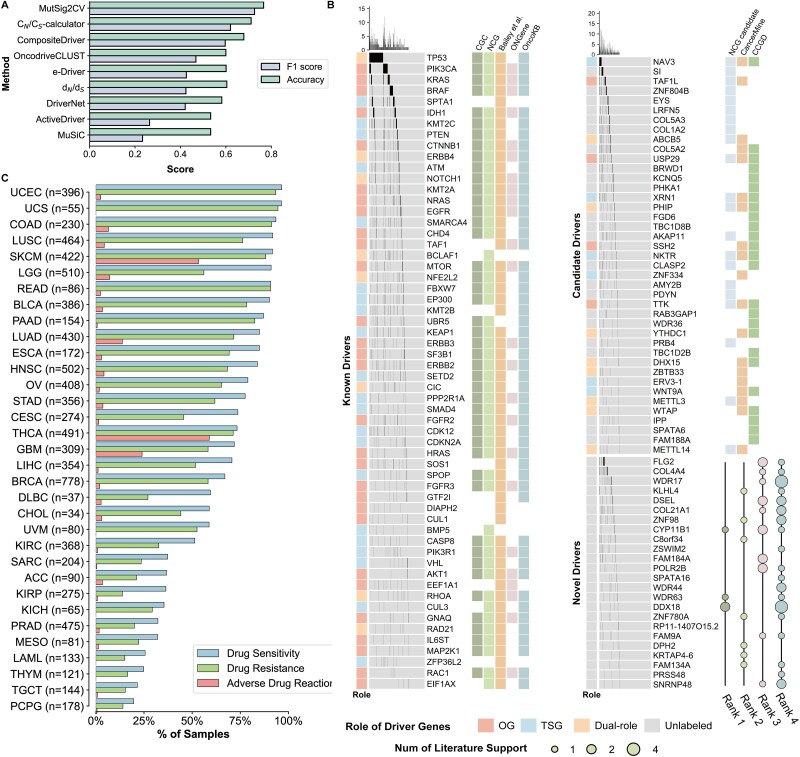
The evaluation of genes under significant positive selection. (A) The F1 score and accuracy of the driver genes predicted by C_N_/C_S_-calculator and other eight driver gene prediction methods. (B) The driver genes identified by C_N_/C_S_-calculator are supported as the known, candidate and novel drivers by multiple levels of evidence. The genes in the left column overlap with a collection of known cancer genes (*see*  *Methods*), and are thus referred to as “known drivers.” The genes in the upper right column overlap with a collection of candidate cancer genes (*see*  *Methods*), and are thus referred to as “candidate drivers.” The remaining genes are labeled as “novel drivers.” For each “novel driver,” we manually collected literature evidence for their role in cancer (Supplementary Table S5), where “Rank1” represents direct oncogenic effect, “Rank2” represents indirect oncogenic effect, “Rank3” represents being a molecular signature of cancer and “Rank4” represents being predicted to drive cancer development. The size of the circle represents the amount of literature evidence. OG, oncogene. TSG, tumor suppressor gene. (C) Percentage of samples with at least one putatively actionable missense mutation in predicted driver genes for each cancer type.

Compared to capturing positive selection using *C_N_/C_S_* metrics alone, *C_N_/C_S_*-calculator showed significantly improved performance in predicting driver genes, second only to MutSig2CV (Fig. 4A). Leveraging multiple evolutionary signatures in a robust framework, our method proved capable of not only retrieving known cancer driver genes but also nominating a rich repertoire of novel putative driver genes.

Among the 129 identified drivers (Supplementary Table S1), the “Filter Dubious Genes” option eliminated six genes (see *Methods*) (Supplementary Table S4), resulting in 123 potential driver genes, of which nearly 50% of the predicted genes are widely recognized “Known Drivers” (see *Methods*) with proven oncogenic or tumor suppressor roles (Fig. 4B, Supplementary Fig. S2), including well-established cancer genes such as *TP53*, *PIK3CA*, and *KRAS*. This percentage was up to 80% when “Candidate Drivers” (see *Methods*) were considered (Supplementary Fig. S3). Particularly, our analysis identified *NAV3* as a promising driver, characterized by a *C_N_/C_S_* value of 1.44 and $\eta$ value of 0.006. Notably, *NAV3* has been consistently categorized as a candidate driver in all three authoritative databases (NCG candidate [46], CancerMine [47], and CCGD [48]), further supporting its potential role in cancer development.

For the remaining 24 genes not cataloged in the database, defined here as “Novel Drivers”, we manually collected literature evidence for their role in cancer (Supplementary Table S5). Results shown that up to 40% (10/24) of novel drivers had direct or indirect experiment evidence for their functions in carcinogenesis, and another nine novel drivers were identified as molecular signatures of cancer, which supports further research on their functions (Fig. 4B). A particularly compelling example is *WDR63*, which emerged from our analysis as a promising driver with *C_N_/C_S_* value of 2.06 and $\eta$ value of 0.004. Subsequent literature validation proved *WDR63* as a transcriptional target of p53, functioning as a negative regulator of cell migration, invasion, and metastasis through its inhibition of Arp2/3-mediated actin polymerization. The knockdown of *WDR63* greatly increased the invasive ability of multiple cancer cell lines [49], supporting it as a potential tumor suppressor gene.

Furthermore, we used data collected from the OncoTriMD [50] database (Supplementary Table S6) to analyze the clinical significance of the identified driver genes and found that nearly 50% (61/123) of the driver genes were clinically actionable for the use of drugs targeting their specific molecular events. For example, silencing of the novel driver *DDX18* in three tamoxifen-resistant cell lines resulted in significant inhibition of growth in the presence of tamoxifen [51], which emphasizes the clinical potential of novel drivers. The clinically actionable drivers covered the majority of cancer patients, especially in UCEC, where 96% (382/396) of patients had mutations in driver genes that may lead to drug sensitivity, resistance, and side effects (Fig. 4C). These findings highlight the potential of the driver genes for targeted therapeutic intervention in a wide range of cancer patients.

### *C_N_/C_S_*-calculator unveiled mini-drivers with multifaceted oncogenic functions

**Figure 5:**
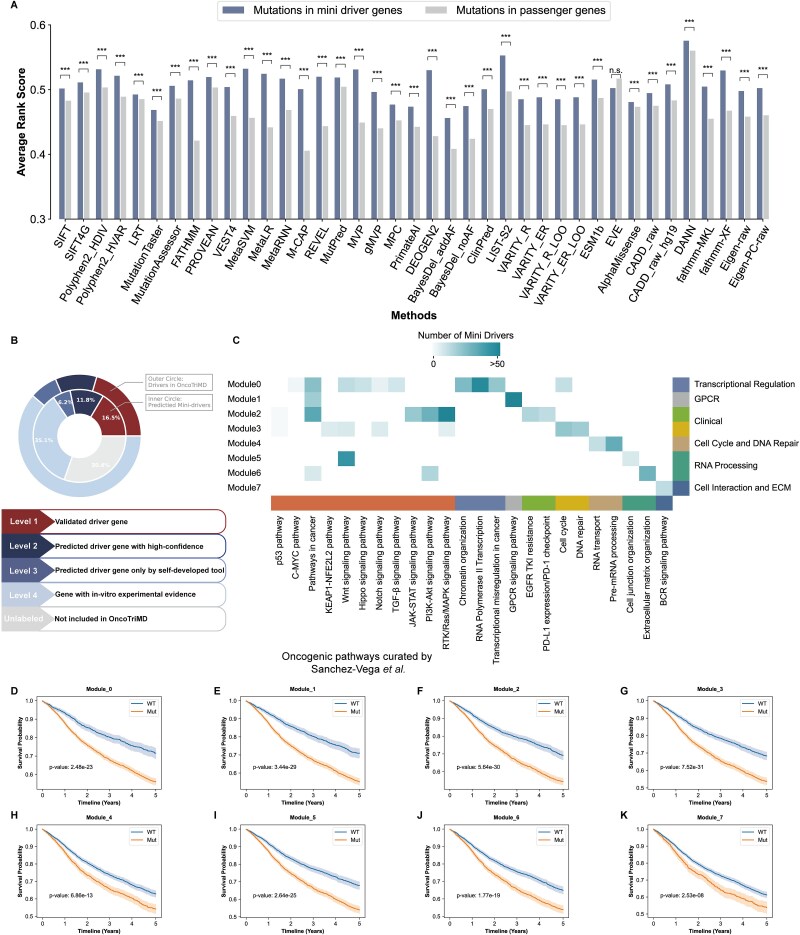
The functional analysis of mini-driver genes at the site (A), gene (B) and module (C and D) levels, respectively. (A) The average rank score of mutations in mini-drivers versus mutations in passenger genes annotated by 34 variant function prediction methods. The larger the score the more likely the mutation has damaging effect. Statistical significance was tested by t test (^*^P < .05; ^**^P < .01; ^***^P < .001; n.s. *P* > .05). (B) The outer circle demonstrates the proportion of different levels of driver genes in the OncoTriMD database. The inner circle demonstrates the overlap between the predicted mini-drivers and the OncoTriMD collected drivers with different levels. (C) Significantly enriched pathways (FDR < 0.05) for eight mini-driver modules. (D-K) The group that was mutated in the mini-driver module had a significantly shorter survival than the group without mutations.

*C_N_/C_S_*-calculator has the advantage of detecting weak positive selection signals that are overlooked by conventional selection pressure analysis, making it a promising tool for identifying mini-driver genes. We comprehensively characterized the molecular functions of the identified 2354 mini-driver genes at the site, gene, and module levels, respectively. At the site level, 33 out of 34 mutation function prediction methods [52] show that the mutated sites in mini-drivers have significantly higher damaging rank scores than sites in passengers (Fig. 5A), indicating the deleterious nature of mutations in mini-drivers. The ClinVar database [53] annotated nearly 500 mutated sites in mini-drivers as “Likely Pathogenic” or “Pathogenic” (Supplementary Table S7) and most of them are not included in any known cancer gene list, highlighting the attention they should be paid in future research. A representative example is *SGK2*, which is identified as a mini-driver with relatively low *C_N_/C_S_* ratio (1-H < *C_N_/C_S_* < 1) but high $\eta$ (0.01) and *Ω_dri_* value (7.46). The R187Q mutation in SGK2 is annotated as “Likely Pathogenic” by ClinVar. Meanwhile, some study have experimentally confirmed that the SGK2 regulates the degradation of oncogenic protein PTOV1 [54]; serves as a synthetic lethality target in p53^−/−^ cells [55–57]; and promotes cancer cell migration and resistance [58–60]. The interplay between mini-drivers and cancer genes suggests that mini-drivers may fine-tune or optimize the side effects of major-driver mutations so that fitness is increased [25].

When compared mini-drivers with the cancer driver genes labeled by different levels of evidence in the OncoTriMD database (where the confidence level of evidence, from high to low, corresponds to genes labeled as Level1–4) (Supplementary Table S8), we observed a substantial overlap (34.5%, 812 out of 2354) between the mini-driver genes and the driver genes labeled as Level1-Level3 (hypergeometric test, with *P values* of Level1 = 4.25 × 10^−4^, *P values* of Level2 = 1.15 × 10^−15^, and *P-values* of Level3 = 6.10 × 10^−3^, respectively) (Fig. 5B). The overlap with different levels of driver genes hints once again at the potential of these weakly positively selected genes as mini-drivers. Moreover, similar to driver genes, these investigated mini-drivers reconcile with the concept of oncogenes and tumor suppressors, with 11.5% of them labeled as oncogenes and 7.3% of them labeled as tumor suppressors (Supplementary Fig. S2, Supplementary Table S2). Mini-drivers can also fill the void of drivers, particularly in understanding why some tumor samples lack drivers, as 90.6% of the “driver-absent” cancer patients can be explained by the mutations in mini drivers. When mutations in driver and mini driver genes are considered together, 98.3% of cancer patients could be covered (Supplementary Fig. S4), suggesting a broad application of these driver and mini driver genes in cancer phenotype identification.

Further, to clarify the interaction between mini-driver genes and known cancer drivers, we projected mini-drivers, passengers, and known drivers onto the Reactome functional interaction (FI) network [61], and found that the average driver/mini-driver connection degree (i.e. the number of interacting driver genes per mini-driver gene) is 8.4, while the driver-passenger connection degree is 4.7. More frequent connections between drivers and mini-drivers suggest that the mini-drivers may converge on the cancer driver-related pathways and work together to promote tumor progression.

We then constructed a mini-driver FI network, applied edge-betweenness network clustering to it, and obtained eight modules had size >20. To explore what biological features the mini-drivers may connote at the module level, we annotated these eight modules using pathway enrichment analysis. Most of the mini-driver modules were significantly enriched in the widely recognized oncogenic pathways curated by Sanchez-Vega *et al.* [62] (FDR < 0.05) (Fig. 5C), suggesting that the study of malignant transformation should be broadened from major cancer genes to other members of known oncogenic pathways. In addition, each of the eight modules perturbs specific cancer-related functional motifs (FDR < 0.05), demonstrating that the predicted mini-drivers could promote cancer progression across multiple aspects, including transcriptional regulation, drug resistance, cell cycle, DNA repair, tumor microenvironment and so on (Fig. 5C). The property of mini-drivers clustering into specific functional modules also explains how mini-drivers contribute to inter-tumor heterogeneity, as diverse mutational profiles of mini-drivers may lead to similar cancer phenotypes. Moreover, patients with mutations in the mini-driver module had a significantly worse prognosis than those without mutations in the mini-driver module (Figs 5D–K), which remained true after correcting for mutation burden, age and gender of the patients (Supplementary Fig. S5). Module-level analyses provide further evidence that mini-drivers can provide functional alterations similar to—or overlapping with—those of a major driver [25], but in an attenuated and cumulative form.

### Exploring micro-effect polygenic model conducted by mini-drivers in cancer evolution

Due to the characteristics of mini-driver genes, it is difficult to verify whether they lead to cancer phenotypes at the single-gene level. Therefore, we demonstrated the additive effects of mini-driver genes by constructing cancer phenotype prediction models (see *Methods*). Briefly, the model quantifies the performance of the aggregated effect of a gene set to infer binarized trait (cancer versus no cancer). We compiled a dataset containing somatic mutation information from cancer patients and healthy individuals (see *Methods*).

**Figure 6:**
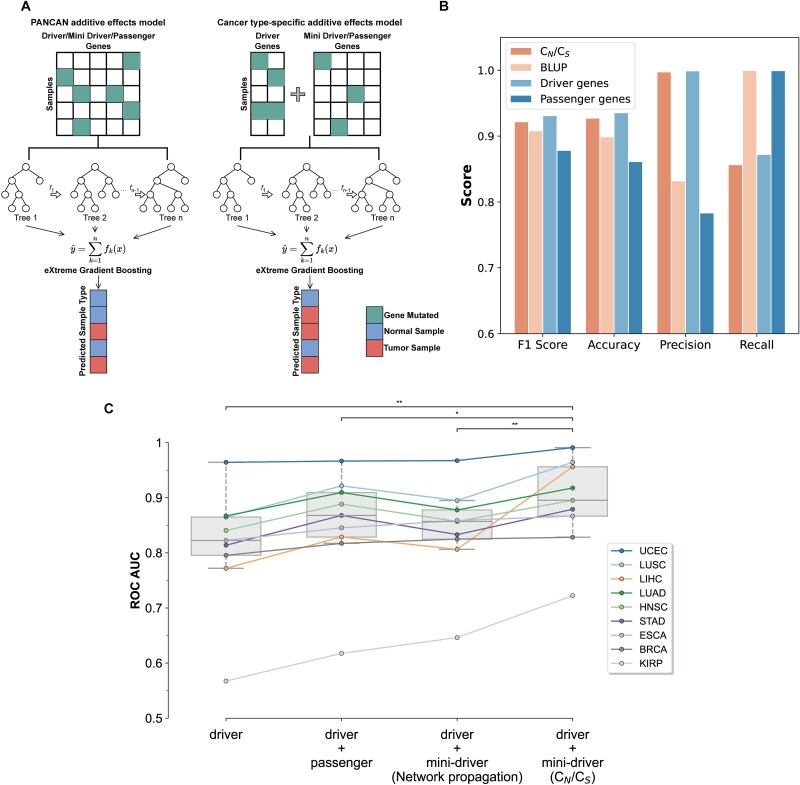
The additive-effects of mini-driver genes. (A) The construction of cancer risk prediction models for pan-cancer mini drivers and cancer type-specific mini drivers, respectively. (B) Validation of the additive effects of mini-driver genes at pan-cancer level. The C_N_/C_S_-calculator-based mini-drivers show additive effects comparable to those of drivers. (C) Validation of the additive-effects of mini-driver genes at cancer type-specific level. The additive-effects of C_N_/C_S_-calculator-based mini-drivers play a complementary or synergistic role to driver genes during cancer progression, as evidenced by the mutation information of mini drivers contributing to the interpretation of cancer phenotypes. Central bar shows median, boxes show interquartile range, and whiskers show range excluding outliers, all over nine cancer types. Statistical significance was tested by *paired samples t-test*.

At the pan-cancer level, we constructed four cancer phenotype prediction models using the eXtreme Gradient Boosting (XGBoost) algorithm [63], each with mutation information of known driver gene, passenger gene, BLUP-based mini-driver gene (predicted by Kumar *et al.* [19] using best linear unbiased estimation method), and *C_N_/C_S_*-calculator-based mini-driver gene lists as input (Fig. 6A). Our analysis revealed that the performance of predicting cancer phenotype using mutation information from *C_N_/C_S_*-calculator-based mini-drivers closely approximated that of known driver genes (Fig. 6B, Supplementary Fig. S6A, C). This finding demonstrates that the additive effects of the identified mini-drivers are comparable to those of known drivers, supporting the hypothesis that although a single mini-driver may exert relatively weak oncogenic effects, their collective impact should not be overlooked. Moreover, the *C_N_/C_S_*-calculator-based model outperformed the BLUP-based [19] and passenger-based models, except for recall (Fig. 6B, Supplementary Figs S6B–D). High recall and low precision scores of the BLUP-based and passenger-based models suggest that the mutation information of these genes is insufficient to distinguish tumor samples from normal samples because it contains tumor-unrelated noise. In addition, to correct mutation burden between models, we normalized the total mutation counts for each sample and each gene and then retrained the models. Results shown that the *C_N_/C_S_*-calculator-based model still demonstrated comparable performance to the driver-based model (Supplementary Fig. S7).

At the cancer-specific level, given the small number of each mini-driver gene set, we evaluated the additive effects by examining to what extent the addition of mutation information of mini-drivers could enhance the performance of the driver-only model (see *Methods*). As shown in Fig. 6C, after adding mutation information of *C_N_/C_S_*-calculator-based mini-drivers, the model can significantly better explain the cancer phenotype than adding mutation information of passenger genes or network propagation-based mini-drivers (predicted by Mohsen *et al.* [64]) in all the cancer types, which suggests that the mini-drivers may play an assisting or complementary role to major drivers. Same as the analysis conducted in pan-cancer level, after correcting the mutation burden, the models with added mutation information of *C_N_/C_S_*-calculator-based mini-drivers still demonstrated the most significant improvement in performance (Supplementary Fig. S8).

### Negative selection uncovers dual role of conserved mini-drivers: essentiality and context-dependent oncogenicity

**Figure 7:**
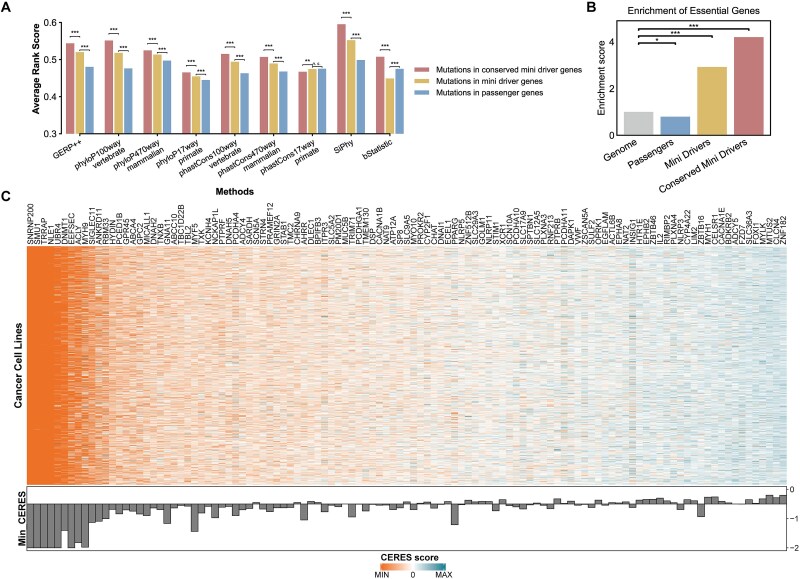
Evolutionary conservation and functional validation of conserved mini-driver genes. (A) The average rank score of mutations in conserved mini-drivers, mini-drivers and passenger genes annotated by nine conservation scores curated in the dbNSFP4.7a. The larger the score the more likely the mutation is evolutionarily conserved. Statistical significance was tested by t test (^***^P < .001; n.s. *P* > .05). (B) Enrichment score of the essential genes curated in the dbNSFP4.7a in conserved mini-drivers, mini-drivers and passenger genes, using all genes in the genome as the control. Statistical significance was tested by χ^2^ test (^*^P < .05, ^***^P < .001). (C) The heatmap shows the CERES scores of 111 conserved mini-driver genes from the Achilles project. The barplot represents the minimum CERES score across all cancer cell lines. A negative CERES score indicates that knocking out the gene inhibits the survival and proliferation of the cell lines.

The detection of negative selection signals has been hampered by the lack of mutation information in negatively selected genes, sequencing noise, and many other reasons. But the site-component specific calculation of *C_N_/C_S_*-calculator skillfully avoids the direct calculation of the number of low-frequency nonsynonymous mutations but provides a statistical method to indirectly estimate the strength of negative selection. This advantage helps us to detect whether the identified mini-drivers further conform to the third major feature, i.e. some sites are under negative selection to maintain essential functions. Accordingly, from the aforementioned mini-drivers, we further identified a particular set of conserved mini-driver genes, which exhibit significantly higher evolutionary conservation scores across multiple methods (Fig. 7A) and are more enriched for essential genes based on dbNSFP4.7a annotations compared with passengers and non-conserved mini-drivers (Fig. 7B). Also, some of the conserved mini-drivers, such as *SMU1*, *MYH9*, *EEFSEC*, etc., have been catalogued as house-keeping genes (Supplementary Table S9), which have constant level of expression across normal tissues [65]. These results support that conserved mini-drivers have maintained evolutionary conservation and may contribute to fundamental cellular processes essential for cell viability.

To further validate the essential function of the conserved mini-drivers, we mapped the conserved mini-drivers to the data collected from the Achilles project [66], a high-throughput screen aimed at identifying essential genes in genomically characterized cancer cell lines. Results shown that, 64% conserved mini-drivers (71 of 111) were considered essential in at least one cell line (CERES scores < −0.5) (Fig. 7). While this proportion is not statistically enriched over the genomic background, it serves to identify a high-confidence subset of conserved mini-drivers whose indispensable role in cancer cell viability is experimentally supported. For instance, we identified *SMU1* as a conserved mini-driver which exhibits high *Ω_dri_* value of 5.08 contrasting with a low *Ω_pass_* value of 0.46. This pattern suggests that while the driver component of *SMU1* potentially exerts context-dependent oncogenicity, its native function appears indispensable for cancer cell survival. Functionally, as a chromatin-binding protein involved in DNA replication regulation and spliceosome activation [67], *SMU1* demonstrates remarkable essentiality, with an average CERES score of −2.63 and is essential in 99.8% (1079/1081) cancer cell lines. Experimental evidence also confirms that *SMU1* knockdown significantly inhibits gastric carcinoma growth, migration, and invasion [68]. Such an essential role of *SMU1* is attributed to the fact that complete loss of function of *SMU1* leads to aberrant DNA replication, alternative RNA splicing and chromosomal instability, which in turn causes cell death [67, 69, 70]. Beyond the essential cellular functions of *SMU1*, somatic mutations in its driver components may confer gain-of-function or fine-tuning through aberrant liquid–liquid phase separation [71] or other yet-to-be-discovered mechanisms. Despite the current lack of evidence for its oncogenic mechanism, the driver component of *SMU1* (p.R346C) was predicted to be deleterious or pathogenic by 15 out of 24 variant function prediction methods (Methods) (Supplementary Table S10), and the damaging rank scores of this driver component is significantly higher than that of sites in passenger genes (*t-test*, *P* = 4.46 × 10^−8^). Building on previous studies that targeting highly conserved genes can yield therapeutic effects comparable to targeting driver mutations [72], we propose that *SMU1* and other conserved mini-drivers represent promising therapeutic targets, particularly for tumors with high inter-tumor heterogeneity or acquired drug resistance, as they target shared vulnerabilities across diverse tumors.

## Discussion

The rapid development of next-generation sequencing technologies and the implementation of numerous cancer genome sequencing projects have facilitated the development of cancer genomics and reshaped the way tumor-related research is conducted [15, 62, 73–78]. Based on the theory of molecular evolution, previous studies have exhaustively analyzed the different selection patterns during cancer evolution and proposed that cancer driver genes were positively selected in cancer genomes. But inter-tumor heterogeneity is higher than previously thought, with many genes not showing significant positive selection. Conventional selection pressure analyses treat all gene sites equally, which can mask intricate selection dynamics, where some sites face negative selection to maintain functional constraints, with weak positive selection at individual sites being masked, resulting in an overall neutral selection.

Compared to conventional methods, we developed a comprehensive pipeline *C_N_/C_S_*-calculator to characterize selection landscapes at the site level. This method can detect weak positive selection signals that were often ignored by conventional methods, positioning it as a valuable tool for identifying mini-driver genes. By employing *C_N_/C_S_*-calculator to analyze cancer genome, we uncovered two types of weakly positively selected genes: the first class is under positive selection at the gene level and has driver component contributing to oncogenic processes, defined as mini-driver genes; and the second class, on the basis of the first class, has functionally conserved sites, thus defined as conserved mini-driver genes. The identified mini-drivers tend to have higher mutational damaging rank scores compared to passenger genes and converge on oncogenic pathways. Additionally, these mini-drivers, while individually subtle in their effects, exhibit additive functional impacts on cancer progression i.e. comparable to, or complementary to, known drivers.

These findings highlight the importance of updating the tumor therapy paradigms, particularly in tumors lacking major drivers. In such scenarios, targeting a single gene may not yield satisfactory results, but multi-targeted therapies at the pathway or regulatory nodes at the cellular network are more likely to be effective. It is consistent with the perspective of holism, which emphases treating the tumor as a whole and studying its internal interactions and connections [79, 80]. Moreover, by leveraging insights into the functional interplay between mini-drivers and major drivers, therapeutic strategies can be refined to exploit vulnerabilities unique to specific molecular subtypes. For example, the inhibition of *SGK2*, a newly identified mini-driver, causes synthetic lethality with p53 dysfunction in cervical cancer cell lines [55, 56]. As the p53-mutant is one of the most common hallmarks of tumorigenesis, the synthetic lethality with *SGK2* loss holds great promise for cancer treatment. Such approaches may not only improve treatment efficacy but also mitigate resistance and recurrence, which are often driven by the changed adaptive potential of tumor subclones.

Nevertheless, our research has some limitations. The prediction of mini-driver genes may yield false positive results. In fact, this disadvantage is due to the nature of mini-driver genes themselves, as their role can only be highlighted by the accumulation of abundant mutations or in specific conditions. Many studies have been conducted to explain the function of somatic mutations from different perspectives, but they all coincidentally corroborate the concept of mini-drivers [20–24]. While false positives remain a challenge, integrating functional information to screen a specific class of genes that provide conditional growth-promoting effects [81] could refine mini-driver predictions and expand their therapeutic relevance. In addition, the current framework of *C_N_/C_S_*-calculator primarily focuses on detecting mini-drivers based on somatic mutation data and may fail to capture carcinogenic factors driven by nonmutational mechanisms, such as epigenetic, transcriptional, or post-translational alterations [82–84]. Identifying these nonmutational drivers requires a shift from somatic mutation-based analyses towards integrative multi-omics strategies. For instance, nonmutational drivers can be identified by searching for recurrent, aberrant patterns of epigenetic or transcriptional alterations that exhibit weak but consistent selective advantages across tumors [82, 85, 86], particularly when these changes are correlated with the dysregulation of cancer-associated genes. Another promising strategy to address this challenge lies in the development of virtual cell models which have the potential to systematically learn and model the complex, nonlinear mechanisms of how the genome and epigenome regulates gene expression and downstream phenotype [87]. By modeling the intricate regulatory networks that govern behavior of cell and tissue, virtual cell could pinpoint critical dysregulations that function as cancer drivers in the absence of genetic mutations. Ultimately, this systems-level approach promises a more holistic understanding of oncogenesis, paving the way for therapies that target the full spectrum of cancer drivers.

## Materials and methods

### Data collection and preprocessing

**(1) Somatic mutation data.**

We sourced cancer somatic mutation information spanning 33 different tumor types and involving 10 224 cancer donors from the TCGA PanCanAtlas MC3 project [34] (https://gdc.cancer.gov/about-data/publications/mc3-2017). We also excluded highly mutated samples for subsequent analysis. Hypermutators with a mutation count exceeding Tukey’s outlier condition, i.e. >1.5 times the interquartile range above the third quartile in their respective cancer types (3Q + 1.5 × IQR) and have mutations greater than 1000 were excluded. The filtered dataset consisted of 9078 samples with 793 694 missense mutations. The mutation data of normal samples were extracted from the 1000 Genomes Project (phase 3 on GRCh37) (https://www.internationalgenome.org/data-portal/data-collection/phase-3), which sequenced the genomes of 3115 healthy people. In this study, only rare human protein-coding variants (minor allele frequency < 0.05%) were retained.

**(2) Sequences and annotations of human genes.**

Sequences and annotations of human genes were extracted from the Ensembl database (http://www.ensembl.org, GRCh37, Release 75) [88]. For each gene, the transcript most commonly used in TCGA was selected for subsequent analyses.

**(3) Comprehensive compilation of cancer genes, passenger genes and housekeeping genes.**

We collected 578 Tier 1 cancer genes from the COSMIC Cancer Gene Census (v95) [89], 803 oncogenes from the ONGene database [90], 711 known cancer genes from the NCG database (v6.0) [46], 1064 cancer genes from the OncoKB database [91] and 299 cancer driver genes reported by Bailey *et al.* [32]. The driver gene list, which contains 1749 known cancer genes, was generated by concatenating the above lists. This gene list was used as the benchmark “known driver genes” to evaluate our method’s performance.

The candidate drivers were derived from the NCG candidate cancer genes [46], the CancerMine database [47], and the Candidate Cancer Gene (CCGD) database [48].

To generate a list of passenger genes, potentially cancer-related genes were recursively removed from all genes, such as genes included in the driver gene list, genes associated with cancer pathways in the KEGG database (https://www.genome.jp/kegg/), genes present in the Online Mendelian Inheritance in Man (OMIM) database (https://omim.org/), MutSigdb [92] predicted genes associated with cancer and genes whose expression was associated with cancer gene expression. Accordingly, a passenger gene list containing 2144 genes that are most likely not associated with cancer was generated.

The list of human housekeeping genes was obtained from Eisenberg *et al.* [65].

**(4) The functional annotation data of genes.**

The list for the clinical actionability assessment of the driver gene (Supplementary Table S6) and the hierarchical driver gene list for the assessment of mini driver genes (Supplementary Table S8) were obtained from the OncoTriMD database (https://pgx.zju.edu.cn/oncotrimd/).

To collect functional annotations that specify whether each driver/mini-driver gene functions primarily as an oncogene or a tumor suppressor gene, we obtained this annotation from COSMIC Cancer Gene Census (v95) [89], NCG database(v6.0) [46], ONGene database [90], OncoKB database [91], CancerMine database [47] and 299 cancer driver genes reported by Bailey *et al.* [32].

The deleteriousness prediction and functional annotation of mutations were derived from dbNSFP4.7a [52], which compiles prediction scores from 34 algorithms.

The catalog of CERES scores of genes in cancer cell lines is obtained from DepMap portal (22Q4) (https://depmap.org/portal) [66], where CERES is a method for unbiased estimation of gene dependency levels from CRISPR-Cas9 essentiality screens [93].

### Framework of *C_N_/C_S_*-calculator

*C_N_/C_S_*-calculator consists of three modules. With the input cancer somatic mutation information, the first module of *C_N_/C_S_*-calculator computes the selection pressure at the gene level, where *C_N_/C_S_* > 1 denotes gene under positive selection while 1-H < *C_N_/C_S_* < 1 denotes that only several sites are under weak positive selection. Then, for each positively selected gene, the mutational landscape of different amino acid sites is inscribed using a two-component mixture module, which models the proportion of driver component ($\eta$) and passenger component ($1-\eta$) in a gene. Finally, for the site components computed by the second module, the third module calculates site component-specific selection pressure *Ω_driver_* and *Ω_passenger_*, respectively.

Through elucidating the diverse selective pressures acting upon distinct sites within the gene, this method can comprehensively characterize the selective landscape in the cancer genome and guide the identification of cancer characteristic genes, which include driver genes, mini-driver genes and conserved mini-driver genes. Specifically, genes that are subject to significant positive selection at both the gene (*C_N_/C_S_* > 1, ${\chi}^2$ test *P* < .05) and amino acid site levels (*η* > 0, LRT-test *P* < .05), i.e. genes with well-defined driver sites, are defined as driver genes. Genes subject to weak positive selection at only individual sites (1-H < *C_N_/C_S_* < 1, ${\chi}^2$ test *P* < .05) are defined as mini-driver genes. A portion of these mini-driver genes are further defined as conserved mini-drivers when their passenger component are subject to significant negative selection (*Ω_passenger_* < 1, ${\chi}^2$ test *P* < .05).

### *C_N_/C_S_-H* module for the detection of weak positive selection

Because of the strong context-dependence of somatic mutations in cancer, conventional equal-rate methods that assume same probability for each substitution pattern may lead to systematic under- or over-estimation of selection. Therefore, *C_N_/C_S_* ratio corrects biases emerging from sequence context-dependent effects by using an empirical nucleotide mutation model with 96 rate parameters [10]. The *C_N_/C_S_* ratio of a gene is defined by the ratio of the nonsynonymous mutation rate (*C_N_*) to the synonymous mutation rate (*C_S_*) in cancer samples.


(1) \begin{equation*} {\mathrm{C}}_{\mathrm{N}}/{\mathrm{C}}_{\mathrm{S}}=\left(\mathrm{N}/{\mathrm{L}}_{\mathrm{N}}\right)/\left(\mathrm{S}/{\mathrm{L}}_{\mathrm{S}}\right) \end{equation*}

where $N$ or $S$ is the observed number of nonsynonymous or synonymous somatic mutations and ${L}_N$ or ${L}_S$ is the expected number of nonsynonymous or synonymous sites, respectively (see *Supplementary Methods Distinguishing different selection modes in cancer evolution using C_N_/C_S_-H*).

It should be noticed that the estimated *C_N_/C_S_* ratio only measures the mean selection pressure of a gene, casting some doubts about the effectiveness of *C_N_/C_S_* ratio in cancer genomic analyses. Therefore, we proposed *C_N_/C_S_-H* to distinguish different selection modes in cancer evolution more precisely [31]. The definition of *H* is given by Eq.(2), where $E\left[\cdotp \right]$ is short for expectation and $\lambda$ is the evolutionary rate of a nucleotide.


(2) \begin{equation*} H=1-\frac{{\left(E\left[\lambda \right]\right)}^2}{E\left[{\lambda}^2\right]}\kern1em \end{equation*}

As explained in Supplementary Methods Eq.(2)–(10), C_N_/C_S_ values between 1-H and 1 suggests that genes under positive selection with neutral-lethal mode, where mutations can be classified into adaptive mutations, lethal mutations, and neutral mutations. In such a scenario, despite that some functionally important sites in genes are virtually invariable, there are still few sites that confer selective advantage to the cancer cells after mutated (Table 1). This method prevents weak positive selection of individual sites from being obscured by strong negative selection of other conserved sites, and thus can contribute to the identification of mini driver genes. Given the cancer somatic mutation data, the value of $H$ for each gene can be easily calculated as follows:


(3) \begin{equation*} H=\frac{\operatorname{var}(z)-\overline{z}}{\operatorname{var}(z)+\overline{z}\left(\overline{z}-1\right)}\kern1em \end{equation*}

where $z$ is the number of somatic missense mutations at a gene site, $\overline{z}$ is the mean of $z$, and $\mathit{\operatorname{var}}(z)$ is the variance of $z$.

**Table 1:** Relationships between *C_N_/C_S_* and 1-H.

Criteria	Interpretations
*C_N_/C_S_* < 1-H	Nearly neutral evolution, plus some sites under strong selective constraints
*C_N_/C_S_* = 1-H	Neutral evolution, plus some sites under strong selective constraints
1 > *C_N_/C_S_* > 1-H	Positive evolution, plus some sites under strong selective constraints
*C_N_/C_S_* = 1	Neutral evolution virtually in all sites (or a combination between positive and negative selections)
*C_N_/C_S_* > 1	Dominant positive evolution

### Two-component mixture model for determining site components

**(1) Construction of two-component mixture model.**

Inspired by our previous work, CanDriS [94], we developed a two-component mixture model to distinguish between the driver and passenger components within genes. For each gene, this module modeled the number of missense mutations for driver and passenger components separately with Poisson distributions (Supplementary Methods Eq.(11)–(12)).

However, cancer somatic mutations at driver sites are highly recurrent and are usually subject to positive selection that may differ among genes and sites. Therefore, a simple Poisson model might not be sufficient to account for the complexity of driver mutations. We thus developed a more realistic model as follows, which is more applicable to the prediction of driver component (Supplementary Fig. S9, Supplementary Methods). First, at a given driver site, the occurrence of somatic mutations is assumed to follow a Poisson process. Second, ${m}_1$, the recurrence rate of somatic mutations at a driver site, is modeled by ${m}_0+\lambda$, where ${m}_0$ is the recurrent rate at a passenger site and $\lambda$ is a random variable that varies among different driver sites according to a gamma distribution $\varphi \left(\lambda \right)$


(4) \begin{equation*} \phi \left(\lambda \right)=\frac{\beta^{\alpha }}{\varGamma \left(\alpha \right)}{\lambda}^{\alpha -1}{e}^{-\beta \lambda}\kern1em \end{equation*}

where $\alpha$ is the shape parameter, $\beta$ is a scalar, and the mean recurrence rate at the driver sites is ${m}_1={m}_0+\alpha /\beta$.

Let $z$ be the number of somatic missense mutations at a site. It follows that the distribution of $z$ at a driver site is given by


(5) \begin{equation*} {P}_1(z)={\int}_0^{\infty}\frac{{\left({m}_0+\lambda \right)}^z}{z!}{e}^{-\left({m}_0+\lambda \right)}\phi \left(\lambda \right) d\lambda \kern1em \end{equation*}

It is clear that when $\alpha$→∞, ${P}_1(z)$ is reduced to a Poisson model, while when ${m}_1$ >> ${m}_0$, ${P}_1(z)$ approximately follows a negative binomial distribution (NBD), i.e.


(6) \begin{equation*} {P}_1(z)\approx \frac{\varGamma \left(z+\alpha \right)}{z!\varGamma \left(\alpha \right)}{\left(\frac{m_1}{m_1+\alpha}\right)}^z{\left(\frac{\alpha }{m_1+\alpha}\right)}^{\alpha}\kern2.00em \end{equation*}

Therefore, under the Poisson-NBD^*^ model, the distribution of $z$ of the studied gene is given by:


(7) \begin{equation*} f(z)=\left(1-\eta \right)\frac{m_0^z{e}^{-{m}_0}}{z!}+\eta \frac{\varGamma \left(z+\alpha \right)}{z!\varGamma \left(\alpha \right)}{\left(\frac{m_1}{m_1+\alpha}\right)}^z{\left(\frac{\alpha }{m_1+\alpha}\right)}^{\alpha } \end{equation*}

**(2) Implementation of parameter estimations.**

Next, parameter estimation was implemented. The estimation of ${m}_0$ can be seen in (Supplementary Methods Eq.(13)–(14)).

Different shape parameters $\alpha$ make the driver mutations follow different distributions. It is well known that the empirical frequency of somatic mutations occurring in a single gene is usually highly skewed. However, the strategy of estimating the shape parameters for each gene individually is difficult to implement. To overcome this difficulty, we assumed that $\alpha$ was a universal parameter for most cancer genes. After fitting the mutation profiles of the cancer genes to the Poisson-NBD^*^ model using the maximum likelihood (ML) approach, we obtained $\alpha$ = 1.51.

From the cancer somatic mutation data, it is straightforward to calculate the mean and variance of $z$. The following relationships can be derived easily:


(8) \begin{equation*} {\displaystyle \begin{array}{l}\kern2.7pc \overline{z} =\left(1-\eta \right){m}_0+\eta{m}_1\\{}\operatorname{var}(z)-\overline{z} =\eta \left(1-\eta \right){\left({m}_1-{m}_0\right)}^2+\eta \frac{m_1^2}{\alpha}\end{array}}\kern1em \end{equation*}

As long as the shape parameter $\alpha$ is given, we show that the method of moments estimate of ${m}_1$ is given by


(9) \begin{equation*} {m}_1=\left(\frac{\operatorname{var}(z)-\overline{z}}{\overline{z}-{m}_0}+\overline{z}+\frac{m_0}{\alpha}\right)\Big/\left(1+\frac{1}{\alpha}\right)\kern1em \end{equation*}

and the method of moments estimate of $\eta$ is given by


(10) \begin{equation*} \eta =\frac{\overline{z}-{m}_0}{m_1-{m}_0}\kern2.00em \end{equation*}

Finally, an approximate likelihood ratio test (LRT) was designed to assert whether the proportion of the driver component ($\eta$) is significantly larger than 0.

### *C_N_/C_S_*-calculator combining two-component to quantify site-specific selection pressure

Since *C_N_/C_S_* ratio only measures the average effect between cancer-driving sites and passenger sites, it is desirable to develop some new methods that can distinguish the *C_N_/C_S_* ratio between cancer-driving component and passenger component.

In *Section 4.4*, the amino acid sites in a gene have been classified into two groups: cancer-driving component with a probability of *η*, and passenger component with a probability of 1-*η.* Let *Ω_pass_* or *Ω_dri_* be the *C_N_/C_S_* ratio at passenger sites or driver sites of a gene, respectively. It appears that the following relationship between *Ω_pass_*, *Ω_dri_* and the overall *C_N_/C_S_* holds


(11) \begin{equation*} \left(1-\eta \right){\varOmega}_{pass}+\eta{\varOmega}_{dri}={C}_N/{C}_S\kern2.00em \end{equation*}

With a broad range of model conditions, *Ω_pass_* can be estimated by


(12) \begin{equation*} {\varOmega}_{pass}=\frac{S_N/{S}_S}{L_N/{L}_S}\kern2.00em \end{equation*}

where *S_N_* or *S_S_* is the number of nonsynonymous or synonymous sites with one or more somatic mutations, respectively. It is then straightforward to estimate *Ω_dri_* based on Eq.(13), i.e.


(13) \begin{equation*} {\varOmega}_{dri}=\frac{{\left[N/S\right]}^{\ast }}{L_N/{L}_S}\kern2.00em \end{equation*}

where ${\left[N/S\right]}^{\ast }$ is given by


(14) \begin{equation*} {\left[N/S\right]}^{\ast }=\frac{N/S-\left(1-\eta \right){S}_N/{S}_S}{\eta}\kern2.00em \end{equation*}

Finally, under the Poisson-NBD model, the site-specific *C_N_/C_S_* ratio at the $k$-th amino acid site where ${z}_k$ somatic (nonsynonymous) mutations are observed can be calculated by the means of posterior mean, i.e.


(15) \begin{equation*} {\varOmega}_k=\left(1-{Q}_k\right){\varOmega}_{pass}+{Q}_k\left[\frac{z_k+\alpha }{m_1+\alpha}\right]{\varOmega}_{dri}\kern1em \end{equation*}

where ${Q}_k=P\left(\mathrm{driver}|{z}_k\right)$ is given by Supplementary Methods Eq. (15).

### Evaluation of the prediction results of positively selected driver genes

The “Filter Dubious Genes” option [95] compiled a list of suspicious driver genes from the literatures of Lawrence *et al.* [96] and Shyr *et al.* [97] (Supplementary Table S4). The suspicious driver gene list compiled genes that are less likely to be critical for disease development, but are more likely to be assigned diseases-related than expected for protein-coding genes in general. In other words, these genes are recurrent false positives in many mutation-based driver detection methods. Over the past few years, these potentially suspicious genes have already been mistakenly nominated as cancer-associated genes in many published cancer genome studies [98, 99]. Therefore, to enhance the reliability and clinical-usability of our final driver list, we provide this optional operation to exclude these genes [100–102]. Specifically, in this study, we removed six genes: *TTN*, *FLG*, *PCLO*, *CSMD3*, *CNTN5*, *LRP1B*, and their inclusion does not affect the overall evaluation results or conclusions (Supplementary Fig. S10).

The seven algorithms used for comparison can be grouped into four main categories: (i) frequency-based (MuSiC [103], MutSig2CV [104], and OncodriveCLUST [105]), (ii) feature-based, e.g. functional impact (CompositeDriver [32]), (iii) structural (domain)-based (e-Driver [6], ActiveDriver [106]), and (iv) network- or pathway-based (DriverNet [107]). Genes in the driver gene list were labeled as positive samples, while the other genes were labeled as negative samples. The F1-score and accuracy of the predicted results of each algorithm were calculated using the functions provided in the python module “sklearn.metrics” [108].

### Validation of the additive-effects of mini-driver genes

In this study, we demonstrated the additive effects of mini-driver genes by constructing a cancer risk prediction model. If the integrated mutational information of mini-driver genes can accurately distinguish cancer samples from normal samples, then it proves that their additive effects are highly correlated with the development of cancer.

Because there is no recognized list of mini-driver genes, we collected mini-driver genes generated by two pioneering works in this area as a comparison. At the pan-cancer level, Kumar *et al.* [19] predicted 728 mini-driver genes using the best linear unbiased estimation (CI = 95%) (BLUP-based mini-drivers). While at the cancer type-specific level, Mohsen *et al.* [64] identified sets of upward mobility genes (UMGs) in 17 cancer types and proposed that there were weak-drivers contained in UMGs (network propagation-based mini-drivers). In addition, at the pan-cancer level, a known driver gene list and a passenger gene list described in *Methods Data collection and preprocessing (3) Comprehensive compilation of cancer genes, passenger genes and housekeeping genes* were used as benchmarks, while at the cancer type-specific level, the driver genes predicted by Bailey *et al.* [32] and a passenger gene list were used as benchmarks.

To validate the pan-cancer mini-driver genes, we extracted the mutation information of the driver gene list, passenger gene list, and two pan-cancer mini-driver gene lists from the tumor samples and normal samples described in *Methods Data collection and preprocessing (1) Somatic mutation data.* Next, the mutation information of the four lists was converted into sample-gene matrices respectively, and the matrices were split into training sets and test sets. Finally, cancer risk prediction models were constructed using the eXtreme Gradient Boosting (XGBoost) algorithm [63] with the training sets as input. The f1-score, accuracy, precision, recall, area under the ROC curve and confusion matrix were measured to evaluate the ability of the cancer risk prediction models to distinguish tumor samples from normal samples.

In the validation of the cancer type-specific mini-driver genes, nine cancer types that met the following criteria were included: (i) the number of type-specific tumor samples was larger than 100; (ii) had cancer type-specific driver genes predicted by Bailey *et al.* [32]; and (iii) had at least 20 mini-driver gene predicted by the *C_N_/C_S_*-calculator and Mohsen *et al.* [64]. Considering the relatively small number of each gene set, we evaluated the additive-effects of mini-driver genes by examining the extent to which the addition of mini-driver information can enhance the performance of the driver-only models. Specifically, four models were constructed for each cancer type: the first model utilized mutation information of driver genes only; the second model utilized mutation information of driver genes and *C_N_/C_S_*-calculator-based mini-drivers; the third model utilized mutation information of driver genes and network propagation-based mini-drivers [64]; and the fourth model utilized mutation information of driver genes and passenger genes as a baseline control. Here, the passenger genes were randomly selected from the passenger gene list with the same number of the C_N_/C_S_-calculator-based mini-drivers. The area under the ROC curve was measured to evaluate the performance of the models.

### Phylostratigraphic analysis

The analysis consisted of the following steps: selecting species for balanced representation, retrieving sequences, building databases, inferring phylostrata, and returning diagnostics. The default UniProt proteome for human was replaced with the FASTA document downloaded from UniProtKB on release 202 303 [109]. We added 102 species to ensure the coverage of each phylostratum. A total of 19 348 human genes were mapped to a phylogenetic tree, including 27 phylostrata, ranging from cellular organisms (Phylostratum 1) to *Homo Sapiens* (Phylostratum 27). Human genes with orthologs in primitive unicellular species were assigned to older phylostrata (Phylostratum 1–3 and taxonomy of cellular organisms, eukaryota, and opisthokonta) and referred to as unicellular (UC) genes. Human genes with orthologs in EM species (Phylostratum 4–14 and taxonomy of metazoa, eumetazoa, bilateria, deuterostomia, chordata, vertebrata, gnathostomata, euteleostomi, sarcopterygii, tetrapoda, amniota) were referred to as EM genes. Human genes with orthologs in mammal species were assigned to younger phylostrata (Phylostratum 15–27 and taxonomy of mammalia, theria, eutheria, boreoeutheria, euarchontoglires, primates, haplorrhini, simiiformes, catarrhini, hominoidea, hominidae, homininae, and *homo sapiens*) and referred to mammal-specific (MM) genes.

**Key Points**

- Developed a selective pressure analysis method *C_N_/C_S_*-calculator to characterize site-level selection landscapes, especially for weak positive selection missed by conventional methods.
- *C_N_/C_S_*-calculator identifies mini-driver genes that exhibit subtle yet additive oncogenic effects, which is comparable to, or complementary to known driver genes in explaining cancer phenotype.
- Our findings highlight a polygenic model of tumor progression, emphasizing the urgent to update tumor therapy paradigms toward multi-targeted strategies, particularly for tumors lacking major drivers.

## Supplementary Material

Supplementary_Information_revision1_bbaf694

Supplementary_Table_bbaf694

Supplementary_materials_bbaf694

## Data Availability

The source code of the *C_N_/C_S_*-calculator is freely available at https://github.com/zjupgx/CNCS-calculator.
