# Supplementary material for: Identification of cancer mini-drivers by deciphering selective landscape in the cancer genome
Source: Brief Bioinform. 2026 Jan 9;27(1):bbaf694. doi: 10.1093/bib/bbaf694 (PMC12784965; doi:10.1093/bib/bbaf694)
Supplement: Supplementary_Information_revision1_bbaf694 [file supplementary_information_revision1_bbaf694.docx]

## Supplementary Information of

**Identification of Cancer Mini-Drivers by Deciphering Selective Landscape in the Cancer Genome**

## Supplementary Methods

### Empirical mutation profile model for *C_N_/C_S_* ratio

*C_N_/C_S_* ratio corrects biases emerging from sequence context-dependent effects by dividing the observed nonsynonymous to synonymous somatic mutations ratio ($N$/$S$) by the expected nonsynonymous to synonymous somatic mutations ratio ($L_{N}$/$L_{S}$). The calculation of $L_{N}$ and $L_{S}$ based on a empirical nucleotide mutation model with 96 rate parameters. For instance, in the codon TTT (coding for amino acid Phe), the first two positions are counted as nonsynonymous sites because no synonymous changes can occur at these positions. At the third position, the transition change (T > C) is synonymous, whereas the remaining two transversion changes (T > A and T > G) are nonsynonymous. Apparently, the weight of the third position of codon TTT as synonymous ($w_{S}$) or nonsynonymous ($w_{N}$) depends on the pattern of somatic mutations.

Denote by $xPy$ the trinucleotide string of any position with base $P$, where the two immediate neighbor nucleotides as $x$, $y$. Since base $P$ has six base-change patterns (under Watson-Crick pairing) and both $x$ and $y$ have four types of bases, there are a total of 4 × 6 × 4 = 96 substitution classifications, with the empirical profile denoted by $M\left( xPy>xP_{i}y \right)$, where $P_{i}$ (i = 1, 2, 3) for the other three bases instead of $P$. To determine the probability of the mutation type ($xPy>xP_{i}y$), we divided the number of mutations in that trinucleotide context ($xPy>xP_{i}y$) by the number of occurrences of the trinucleotide ($xPy$).

Our computational pipeline is illustrated by the following example. In the encoding sequence with two codons …TTTATG…, we consider the third position of codon TTT (Phe). Under the trinucleotide TTA for the mutation profile (not the codon), the corresponding three substitution configurations are given by $M\left( TTA>TCA \right)$, $M\left( TTA>TAA \right)$ and $M\left( TTA>TGA \right)$, respectively, and the number of occurrences of TTA is $M\left( TTA \right)$. Next, we consider codon TTT. Because TTT and TTC are synonymous codons but TTA and TTG are not, the probabilities that this site will be synonymous and nonsynonymous are simply given by the following:

$$\begin{matrix} w_{S} & =M\left( TTA>TCA \right)/M\left( TTA \right) \\ w_{N} & =\left( M\left( TTA>TGA \right)+M\left( TTA>TAA \right) \right)/M\left( TTA \right) \end{matrix} \left( 1 \right)$$

Then, the mutation profiles were depicted as the mutation rate of each mutation type according to the 96 substitution classifications.

### Distinguishing different selection modes in cancer evolution using *C_N_/C_S_*-H

In the theory of molecular evolution, the evolutionary rate ($\lambda$) of a nucleotide is given by

$$\lambda=v\frac{4N_{e}s}{1-e^{-4N_{e}s}} \left( 2 \right)$$

where $\nu$ represents the mutation rate, $s$ represents the coefficient of selection and $N_{e}$ represents the effective population size. Eq. (2) provides a solid foundation for the debate between adaptive evolution, neutral evolution, and nearly neutral evolution: it predicts $\lambda/v>1$ for adaptive evolution ($s$ > 0), $\lambda/v=1$ for neutral evolution ($s$ = 0), or $\lambda/v<1$ for deleterious evolution ($s$ < 0). Usually, $S=4N_{e}s$ is called the selection intensity that uniquely determines the evolutionary rate–mutation rate ratio $\lambda/v$.

Assuming that synonymous mutations are selectively neutral, the *C_N_/C_S_* ratio can be used as a proxy of the $\lambda/v$ ratio. However, each gene site may be subject to different selection intensities ($S$), but the *C_N_/C_S_* ratio is a mean value estimated from all nucleotide sites of an encoding gene. Without loss of generality, we assume that *S* varies among sites according to a distribution $\Phi\left( S \right)$. It follows that the *C_N_/C_S_* ratio is expected to be

$$\frac{C_{N}}{C_{S}}\sim\frac{E\left[ \lambda\right]}{v}=\int\left[ \frac{S}{1-e^{-S}} \right]\Phi\left( S \right)dS \left( 3 \right)$$

where $E\left[ \cdot\right]$ is short for expectation. Noting that the conventional *C_N_/C_S_* tests were actually focused on the mean evolutionary rate-mutation ($\lambda/v$) ratio, which is insufficient to distinguish between different selection scenarios especially when *C_N_/C_S_* < 1. Thus, we consider the second moment of the evolutionary rate, that is,

$$\frac{E\left[ \lambda^{2} \right]}{v^{2}}=\int\left[ \frac{S}{1-e^{-S}} \right]^{2}\Phi\left( S \right)dS \left( 4 \right)$$

Next, a new quantity $\Delta$ is defined as the difference between the second moment and the mean of evolutionary rate, scaled by the mutation rate. From Eq. (3) and (4), it is given by

$$\Delta=\frac{E\left[ \lambda^{2} \right]}{v^{2}}-\frac{E\left[ \lambda\right]}{v}=\int\left( \frac{S}{1-e^{-S}} \right)\left( \frac{S}{1-e^{-S}}-1 \right)\Phi\left( S \right)dS \left( 5 \right)$$

According to the derivation performed by Gu et al.[1], under the classical neutral-lethal mode [2], mutations can be classified into strictly neutral and lethal mutations, at which time $\Delta$ = 0; under positive selection with neutral-lethal mode, mutations can be classified into adaptive mutations, lethal mutations, and neutral mutations, at which time $\Delta$ > 0; under negative selection with nearly neutral mode, mutations can be classified into nearly neutral mutations, lethal mutations, and neutral mutations, at which time $\Delta$ is always less than 0.

Here we illustrate the scenario when $\Delta$ > 0. Suppose that all mutations of a gene are classified into three categories: adaptive mutations ($S$ > 0 with a probability of $f_{A}$), lethal mutations ($S$ = -∞ with a probability of $f_{L}$), and neutral mutations ($S$ = 0 with a probability of $1-f_{A}-f_{L}$). The distribution of (positive) selection intensity for adaptive mutations is denoted by $\Phi^{+}\left( S \right)$. It follows that the mean and the second moments of the evolutionary rate are given by

$$\begin{matrix} E\left[ \lambda\right]=f_{A}v\int_{0}^{\infty} \left[ \frac{S}{1-e^{-S}} \right]\Phi^{+}\left( S \right)dS+\left( 1-f_{A}-f_{L} \right)v \\ E\left[ \lambda^{2} \right]=f_{A}v^{2}\int_{0}^{\infty} \left[ \frac{S}{1-e^{-S}} \right]^{2}\Phi^{+}\left( S \right)dS+\left( 1-f_{A}-f_{L} \right)v^{2} \end{matrix} \left( 6 \right)$$

Hence, one can easily verify that the $\Delta$-measure given by Eq. (5) can be written as follows:

$$\Delta=f_{A}\int_{0}^{\infty} \left( \frac{S}{1-e^{-S}} \right)\left( \frac{S}{1-e^{-S}}-1 \right)\Phi^{+}\left( S \right)dS>0 \left( 7 \right)$$

which is always larger than 0, regardless of the existence of lethal or neutral mutations.

Then, to relate $\Delta$ with the *C_N_/C_S_* ratio, we invoke the *H*-measure [3], which is defined by

$$H=1-\frac{\left( E\left[ \lambda\right] \right)^{2}}{E\left[ \lambda^{2} \right]} \left( 8 \right)$$

Ranging from 0 to 1, a high value of *H* indicates a high degree of rate variation among amino acid sites, and vice versa. After some rearrangements of Eq. (8), we have

$$\frac{E\left[ \lambda^{2} \right]}{v^{2}}=\frac{\left( E\left[ \lambda\right]/v \right)^{2}}{1-H}=\frac{\left( C_{N}/C_{S} \right)^{2}}{1-H} \left( 9 \right)$$

It follows that the relationship between $\Delta$, *H* and *C_N_/C_S_* is given by

$$\Delta=\frac{C_{N}}{C_{S}}\left( \frac{C_{N}/C_{S}}{1-H}-1 \right) \left( 10 \right)$$

That is, $\Delta$ < 0 indicates *C_N_/C_S_* < 1- *H*, $\Delta$ = 0 indicates *C_N_/C_S_* = 1-*H* and $\Delta$ > 0 indicates *C_N_/C_S_* > 1-*H*. Therefore, rejection of the null hypothesis *C_N_/C_S_* = 1-*H* because of *C_N_/C_S_* > 1-*H* suggests a positive selection, whereas some functionally important sites are virtually invariable. This approach prevents weak positive selection of individual sites from being obscured by strong negative selection of other sites, and thus may contribute to the identification of mini driver genes.

### Two-component mixture model for determining site components

**(1) Construction of two-component mixture model**

Inspired by our previous work, CanDriS[4], we developed a two-component mixture model to distinguish between the driver and passenger components within genes. For each gene, this module modeled the number of missense mutations for driver and passenger components separately with Poisson distributions.

The probability of any site being a driver is $\eta$, whereas that of being a passenger is $1-\eta$. Let $z$ be the number of somatic missense mutations at a site. The distribution of $z$ of the studied gene is given by

$$f\left( z \right)=\left( 1-\eta\right)P_{0}\left( z \right)+\eta P_{1}\left( z \right) \left( 11 \right)$$

where $P_{0}\left( z \right)$ is the distribution of $z$ at a passenger site and $P_{1}\left( z \right)$ is that at a driver site.

The two-component mixture model assumes that the number of somatic mutations at the driver site and passenger site both follows Poisson distribution, that is:

$$\begin{matrix} P_{0}\left( z \right)=m_{0}^{z}e^{-m_{0}}/z! \\ P_{1}\left( z \right)=m_{1}^{z}e^{-m_{1}}/z! \end{matrix} \left( 12 \right)$$

where $m_{0}$ is the recurrent rate of somatic mutations at a passenger site and $m_{1}$ is the recurrent rate of somatic mutations at a driver site.

**(2) Estimation of** $m_{0}$ **under the rare-driver assumption**

High-throughput cancer genomics revealed that for almost all cancer genes, only a very small portion of amino acid sites are cancer-driving, that is, $\eta$ << 1, $m_{0}$ < 1, and $m_{1}$ > 1. As $f_{0}$ is expected to be $f\left( z=0 \right)$, from Eq. (11), we have

$$f_{0}=\left( 1-\eta\right)e^{-m_{0}}+\eta P_{1}\left( 0 \right) \left( 13 \right)$$

Under the assumptions that $m_{1}$ >> $m_{0}$ and $\eta$ is small, the first-order approximation can be used to estimate $m_{0}$, that is,

$$m_{0}\approx-ln\left( f_{0} \right) \left( 14 \right)$$

### Posterior probability for a site being cancer-driving

Let $Q_{k}=P\left( driver|z_{k} \right)$ be the posterior probability of being a driver at the $k$-th site of a gene with $z_{k}$ observed somatic mutations. The Bayesian rule claims the following:

$$Q_{k}=\frac{\eta P_{1}\left( z_{k} \right)}{\left( 1-\eta\right)P_{0}\left( z_{k} \right)+\eta P_{1}\left( z_{k} \right)} \left( 15 \right)$$

### Site-specific posterior mean of somatic mutation rate

The Poisson-NBD* mixture model is flexible for implementing a Bayesian approach to predict site-specific posterior mean of the somatic mutation rate. Recall that the rate of recurrent mutation at a driver site is $m_{0}+\lambda$, while the mean of $P_{1}\left( z \right)$ is given by $m_{1}=m_{0}+\alpha/\beta$. Hence, the posterior density of $\lambda$ given $z$ somatic mutations at a driver site is given by

$$P_{1}\left( \lambda\mid z \right)=\frac{\phi\left( \lambda\right)P_{1}\left( z\mid\lambda\right)}{\int_{0}^{\infty} \phi\left( \lambda\right)P_{1}\left( z\mid\lambda\right)d\lambda} \left( 16 \right)$$

Although the exact result of $P_{1}\left( \lambda\mid z \right)$ is tedious, we derived a useful close form when $m_{1}$ >> $m_{0}$; in this case $P_{1}\left( \lambda\mid z \right)$ approximately follows a gamma distribution, with the mean conditional of $z$ given by

$$E\left[ m_{0}+\lambda\mid z \right]=\left( \frac{z+\alpha}{m_{1}+\alpha} \right)m_{1} \left( 17 \right)$$

Thus, given $z_{k}$ somatic mutations at the $k$-th site of a gene, the posterior mean of recurrent mutations at site $k$ is given by $M_{k}=\left( 1-Q_{k} \right)m_{0}+Q_{k}E\left[ m_{0}+\lambda\mid z_{k} \right]$. Together with Eq. (17), we have

$$M_{k}=\left( 1-Q_{k} \right)m_{0}+Q_{k}\left( \frac{z_{k}+\alpha}{m_{1}+\alpha} \right)m_{1} \left( 18 \right)$$

### Benchmark analysis for two-component mixture model

Before the two-component mixture model was applied for driver component prediction, we compared the performance between the Poisson-NBD* model ($\alpha=1.51$) and the original Poisson-Poisson model ($\alpha=inf$), and compared them with 27 recognized driver mutation prediction algorithms. The prediction scores of the 27 algorithms were extracted from dbNSFP4.7a[5]. Functional annotations of the mutations were derived from three highly complementary benchmark datasets. Three benchmark datasets were employed to evaluate the performance of the two-component mixture model (Figure 2A). Seventy-three somatic mutations with oncogenicity annotation derived from *in vivo* tumor formation assays were downloaded from Kim et al. [6]. Of these, 46 mutations categorized as ‘functional’ were used as positive cases and the remaining 27 mutations were used as negative cases. In addition, we sourced *in vitro* cell viability data for missense mutations from FASMIC v1.6 (http://bioinformatics.mdanderson.org/main/FASMIC)[7]. This dataset encompassed a total of 822 mutations, of which 359 mutations annotated as activating, inactivating, inhibitory, or non-inhibitory were considered as positive cases. The remaining 463 neutral mutations were considered negative cases. Moreover, 3692 missense mutations with functional annotations were downloaded from OncoKB v2.7 (https://www.oncokb.org)[8], of which 2929 oncogenic and likely oncogenic mutations were labeled as positive cases and 763 likely neutral mutations as negative cases. For each benchmark dataset, ROC curves and AUC scores were obtained using the R function ‘roc’ provided in the pROC package [9].

## Reference

1. Gu X. dN/dS-H, a New Test to Distinguish Different Selection Modes in Protein Evolution and Cancer Evolution. J. Mol. Evol. 2022; 90:342–351

2. Kimura M. Evolutionary Rate at the Molecular Level. Nature 1968; 217:624–626

3. Gu X. Evolutionary Framework for Protein Sequence Evolution and Gene Pleiotropy. Genetics 2007; 175:1813–1822

4. Zhao W, Yang J, Wu J, et al. CanDriS: posterior profiling of cancer-driving sites based on two-component evolutionary model. Brief. Bioinform. 2021; 22:bbab131

5. Liu X, Li C, Mou C, et al. dbNSFP v4: a comprehensive database of transcript-specific functional predictions and annotations for human nonsynonymous and splice-site SNVs. Genome Med. 2020; 12:103

6. Kim E, Ilic N, Shrestha Y, et al. Systematic Functional Interrogation of Rare Cancer Variants Identifies Oncogenic Alleles. Cancer Discov. 2016; 6:714–726

7. Ng PK-S, Li J, Jeong KJ, et al. Systematic Functional Annotation of Somatic Mutations in Cancer. Cancer Cell 2018; 33:450-462.e10

8. Chakravarty D, Gao J, Phillips S, et al. OncoKB: A Precision Oncology Knowledge Base. JCO Precis. Oncol. 2017; 1–16

9. Robin X, Turck N, Hainard A, et al. pROC: an open-source package for R and S+ to analyze and compare ROC curves. BMC Bioinformatics 2011; 12:77

## Supplementary Figures

**
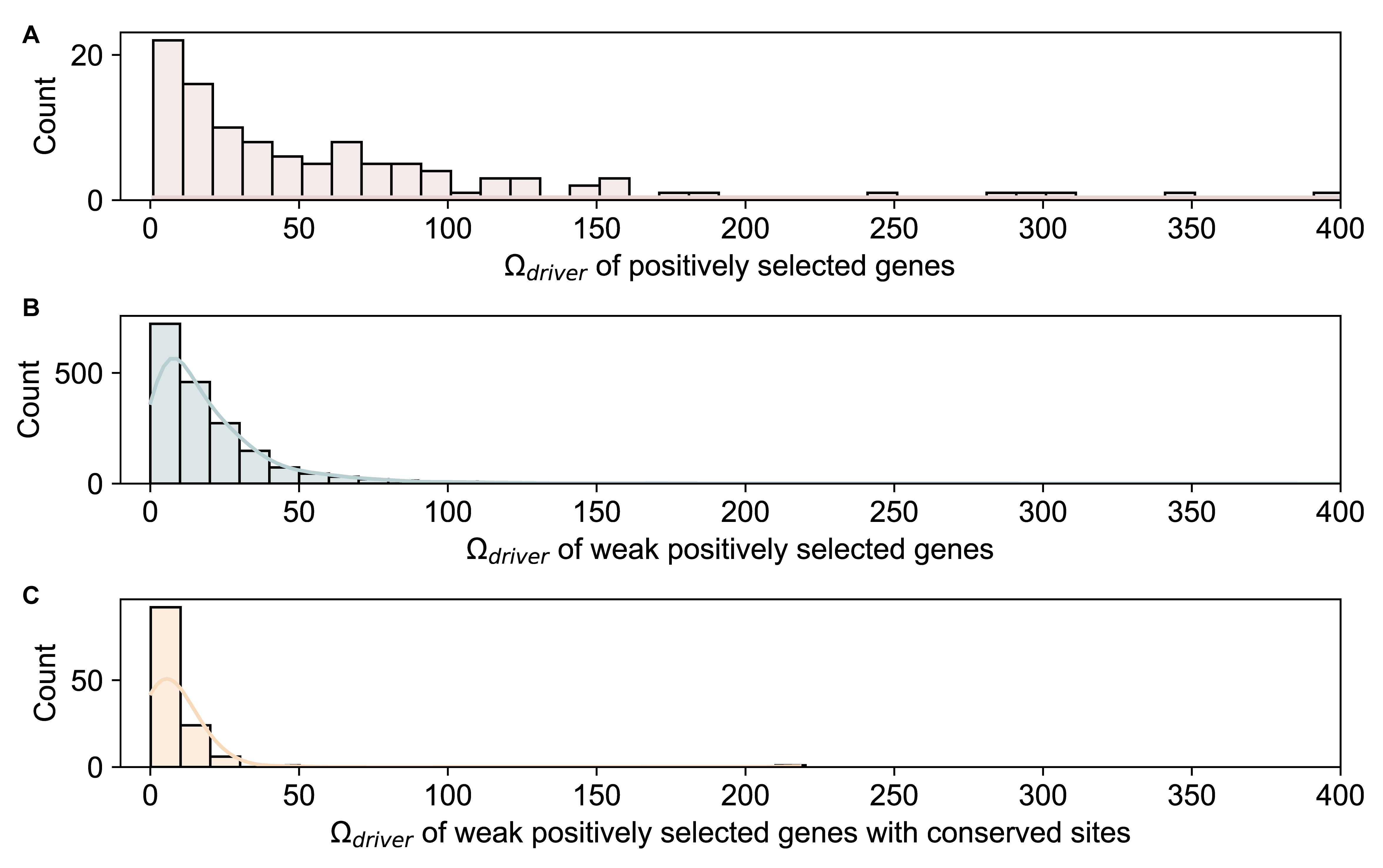
**

**Figure S1.** Distribution of Ω_driver_ values of positively selected genes (A), weakly positively selected genes (B), and weakly positively selected genes with conserved sites(C).


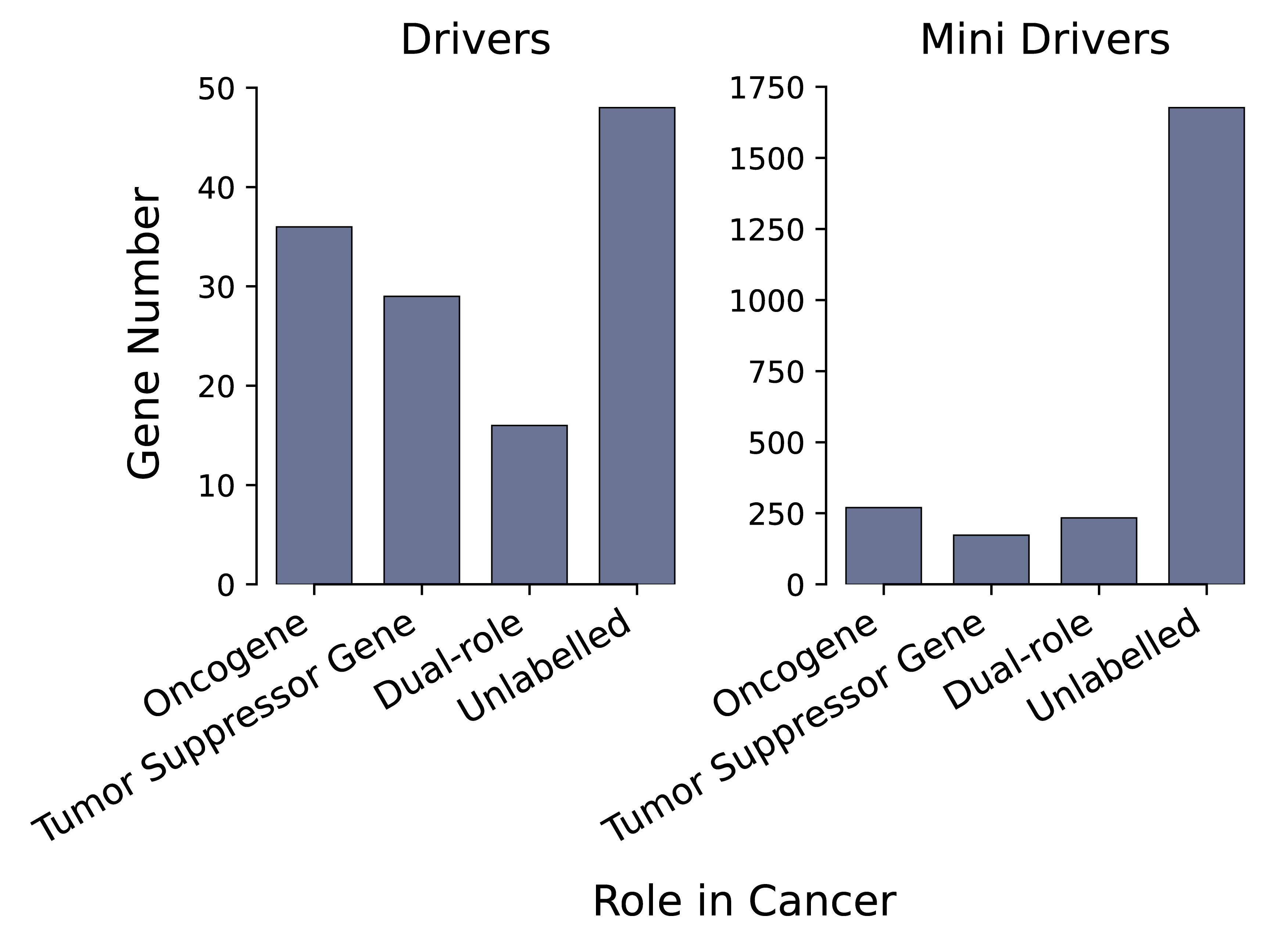


**Figure S2.** Functional annotation of driver and mini-driver genes as oncogene or tumor suppressor gene. If a gene is retrieved with both oncogene and tumor suppressor gene annotations, it will be labeled as ‘Dual-role’.


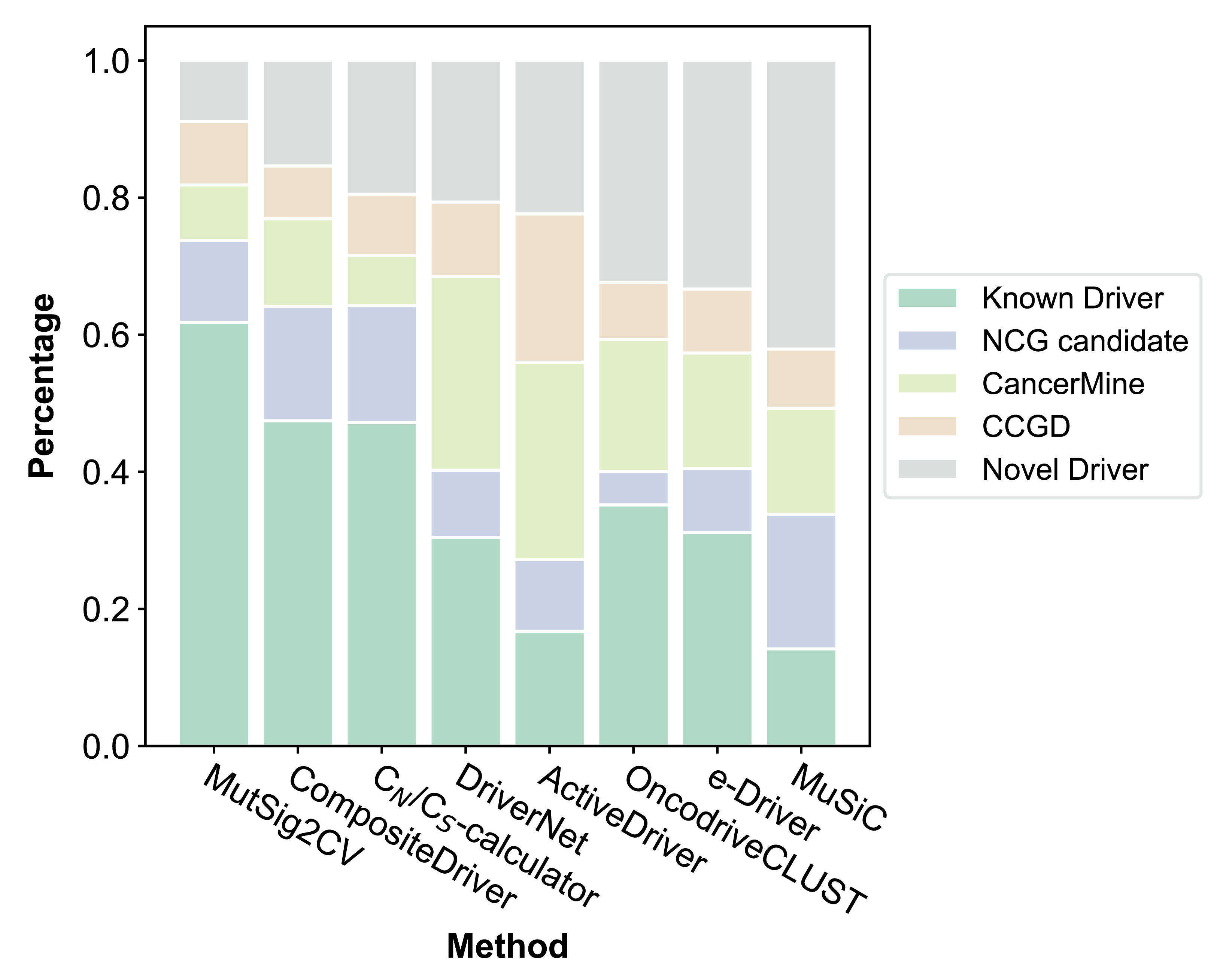


**Figure S3.** The analysis of cancer driver genes predicted by *C_N_/C_S_*-calculator and other seven methods. Nearly 50% of the *C_N_/C_S_*-calculator predicted driver genes were proven to be known drivers, while this value was up to 80% when taking candidate cancer drivers into consideration.


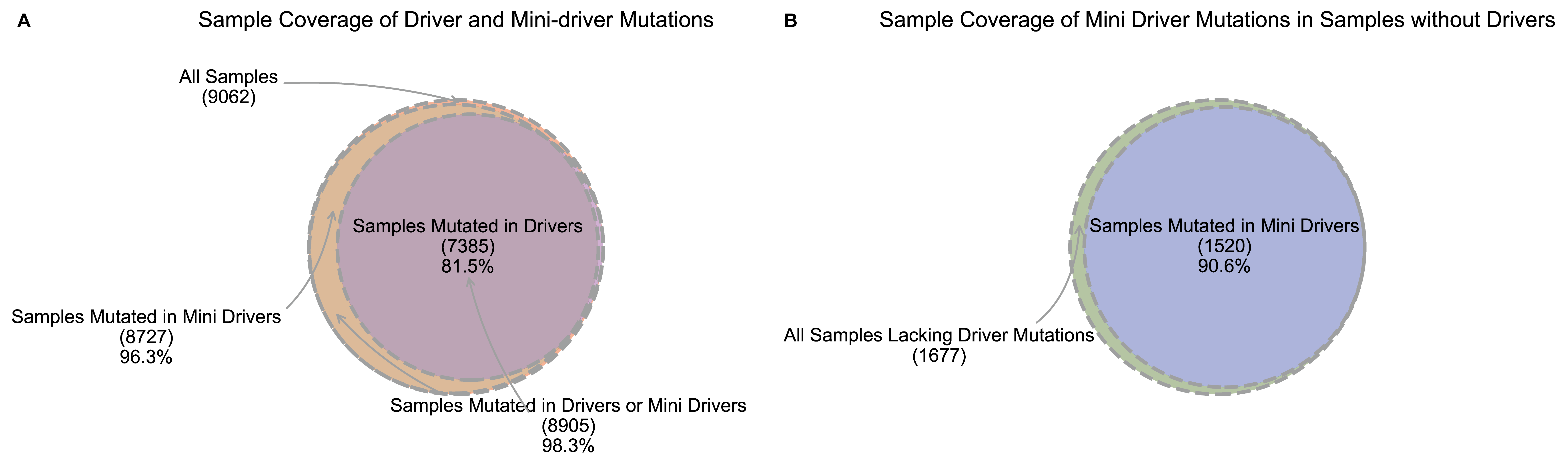


**Figure S4.** (A) The sample coverage of mutations in driver genes and mini driver genes. (B) The sample coverage of mutations in mini driver genes when driver mutations are absent.


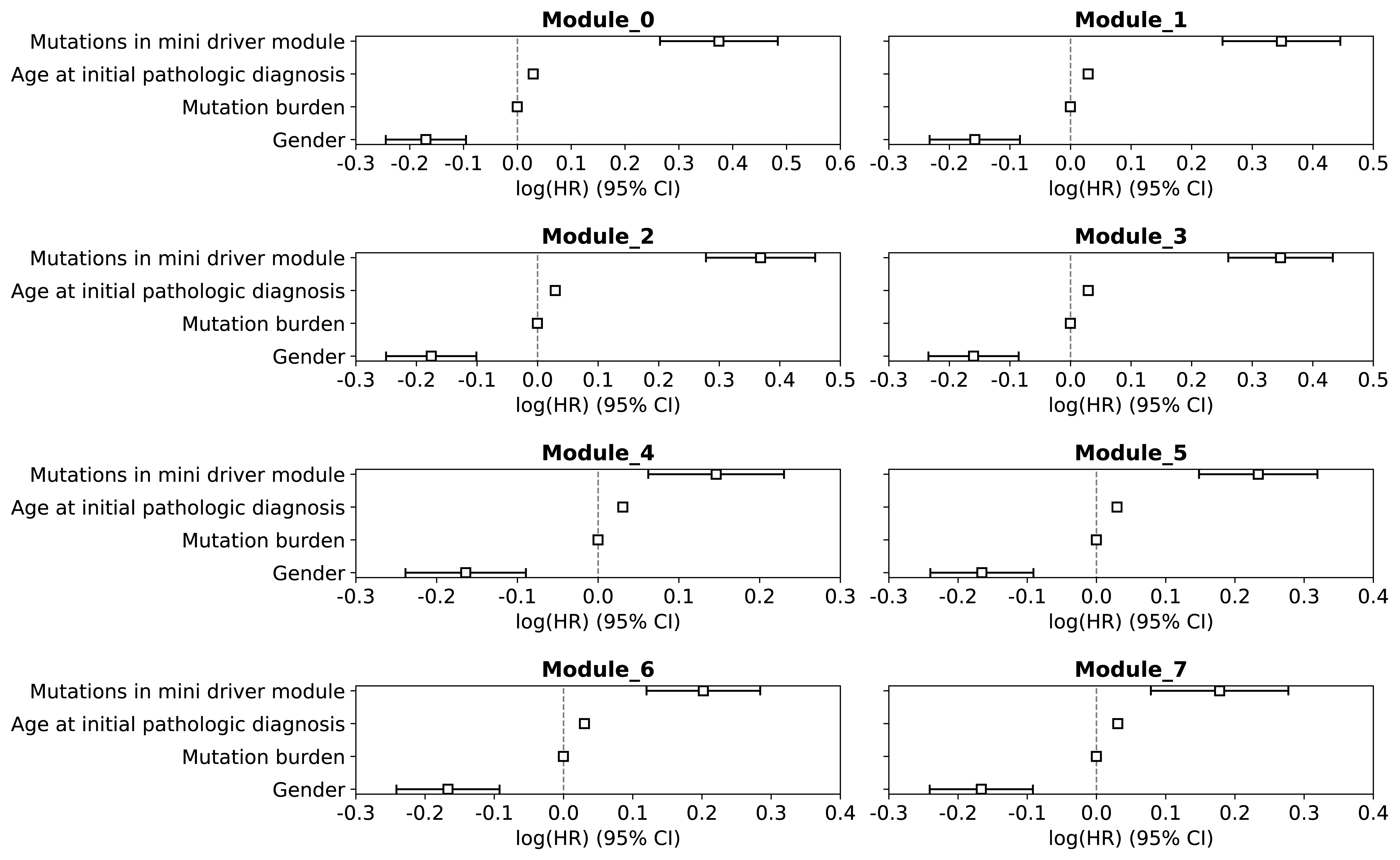


**Figure S5.**  Cox’s proportional hazard models for eight mini-driver modules, which correct mutation burden, age and gender of the patients.


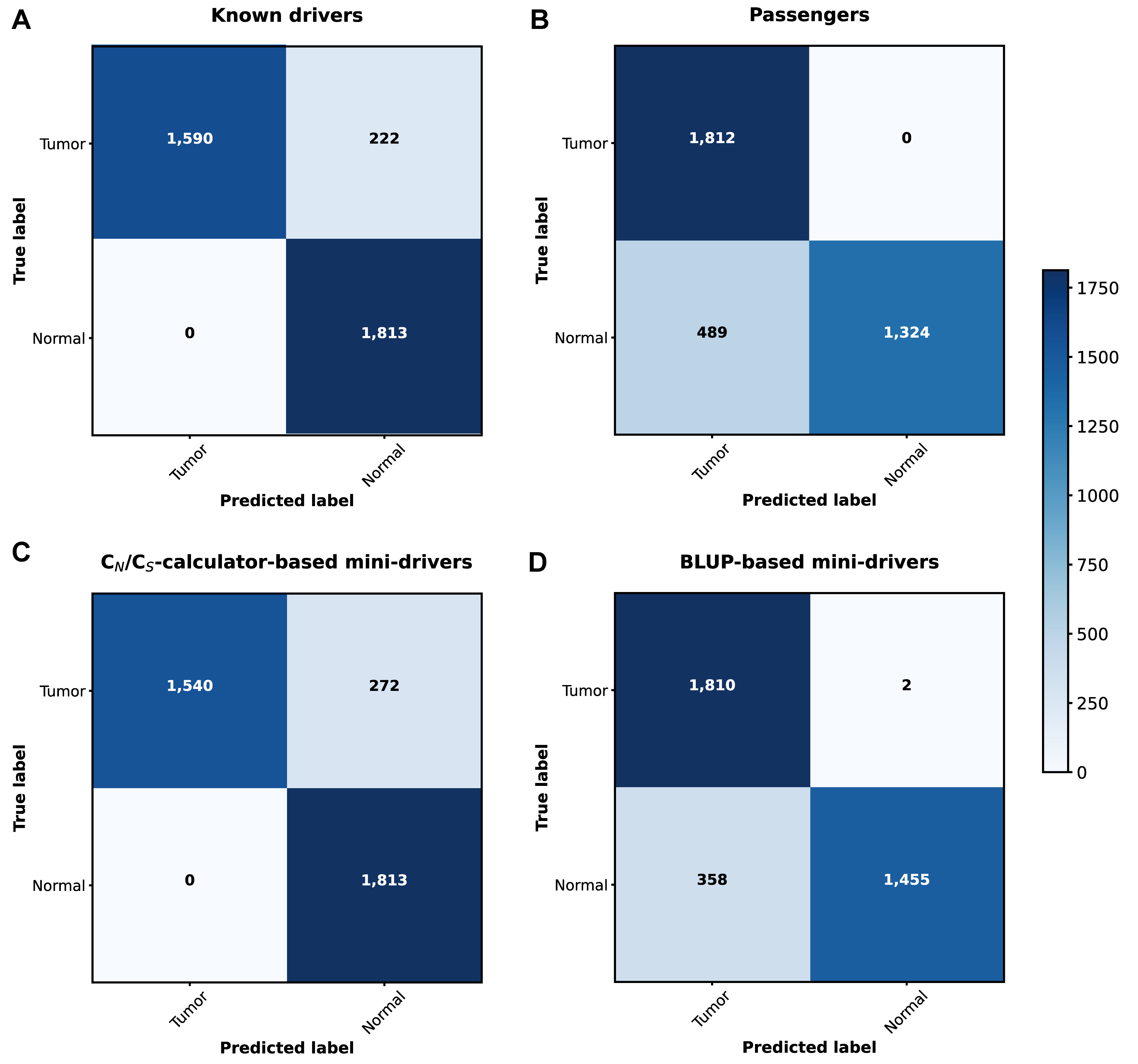


**Figure S6.** Confusion matrices of four cancer risk prediction models constructed with the mutation information of gene lists in Figure 5B.


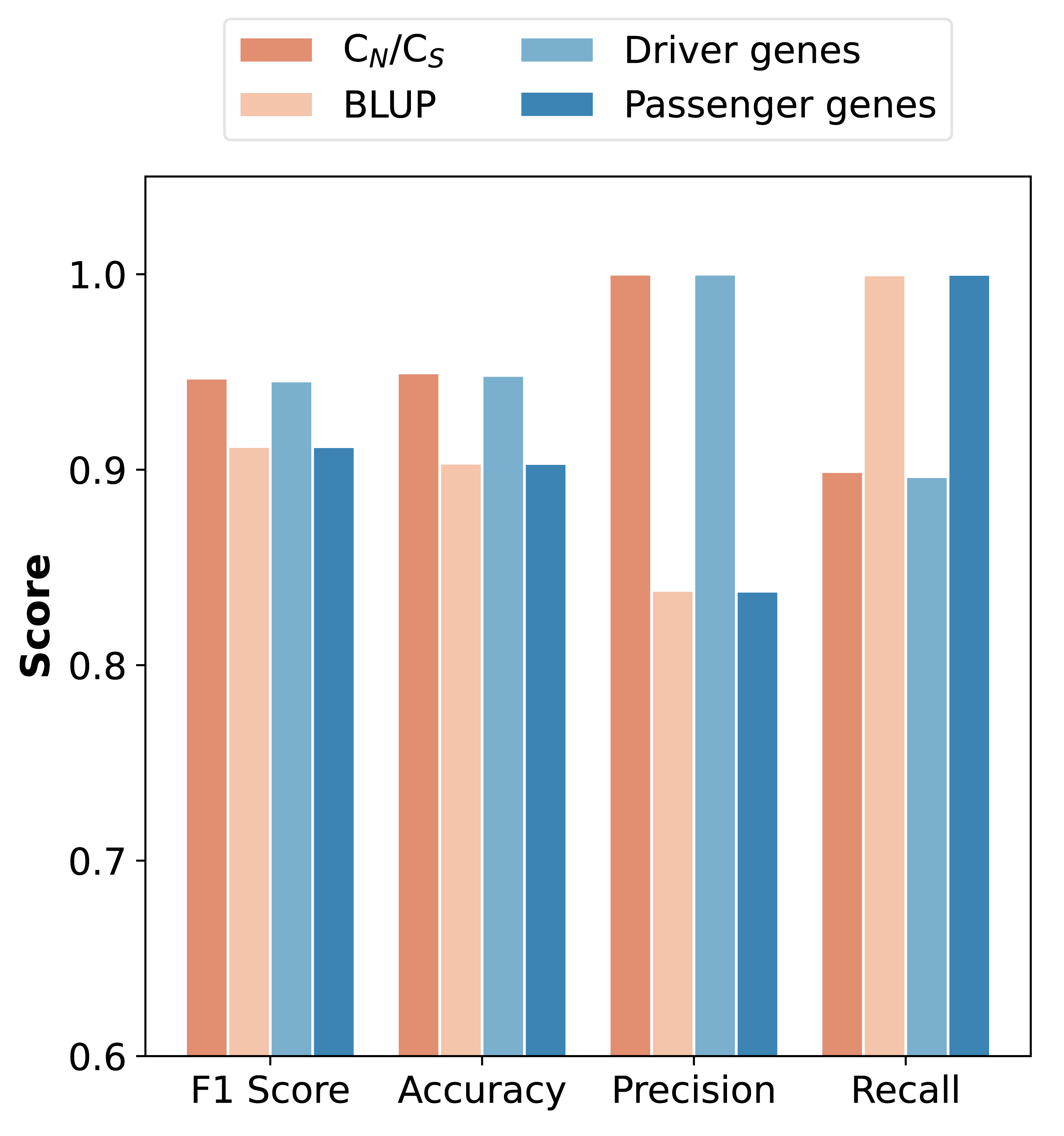


**Figure S7.** Validation of the additive-effects of mini-driver genes at pan-cancer level after correcting mutation burden between models.


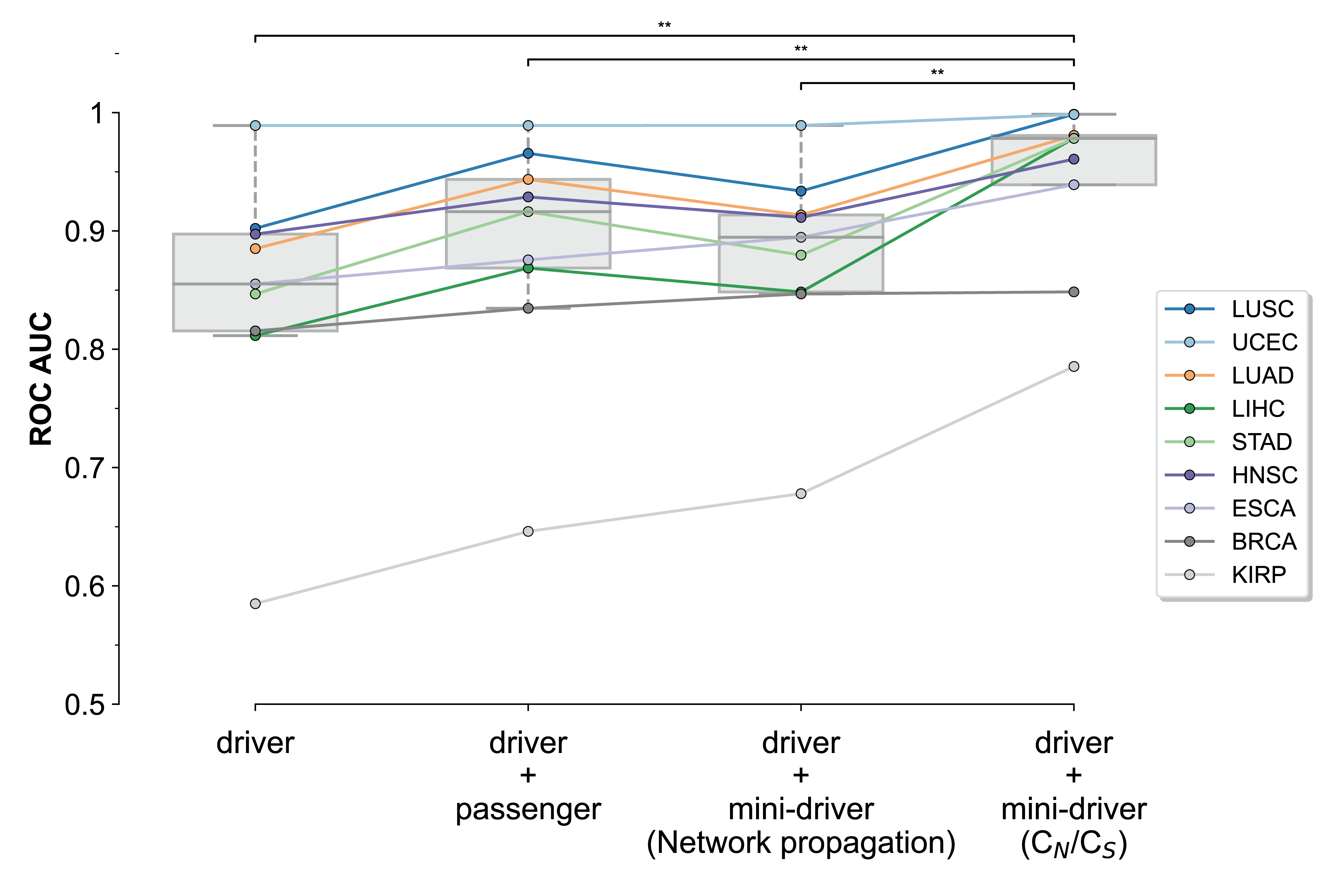


**Figure S8.** Validation of the additive-effects of mini-driver genes at cancer type-specific level after correcting mutation burden between models.


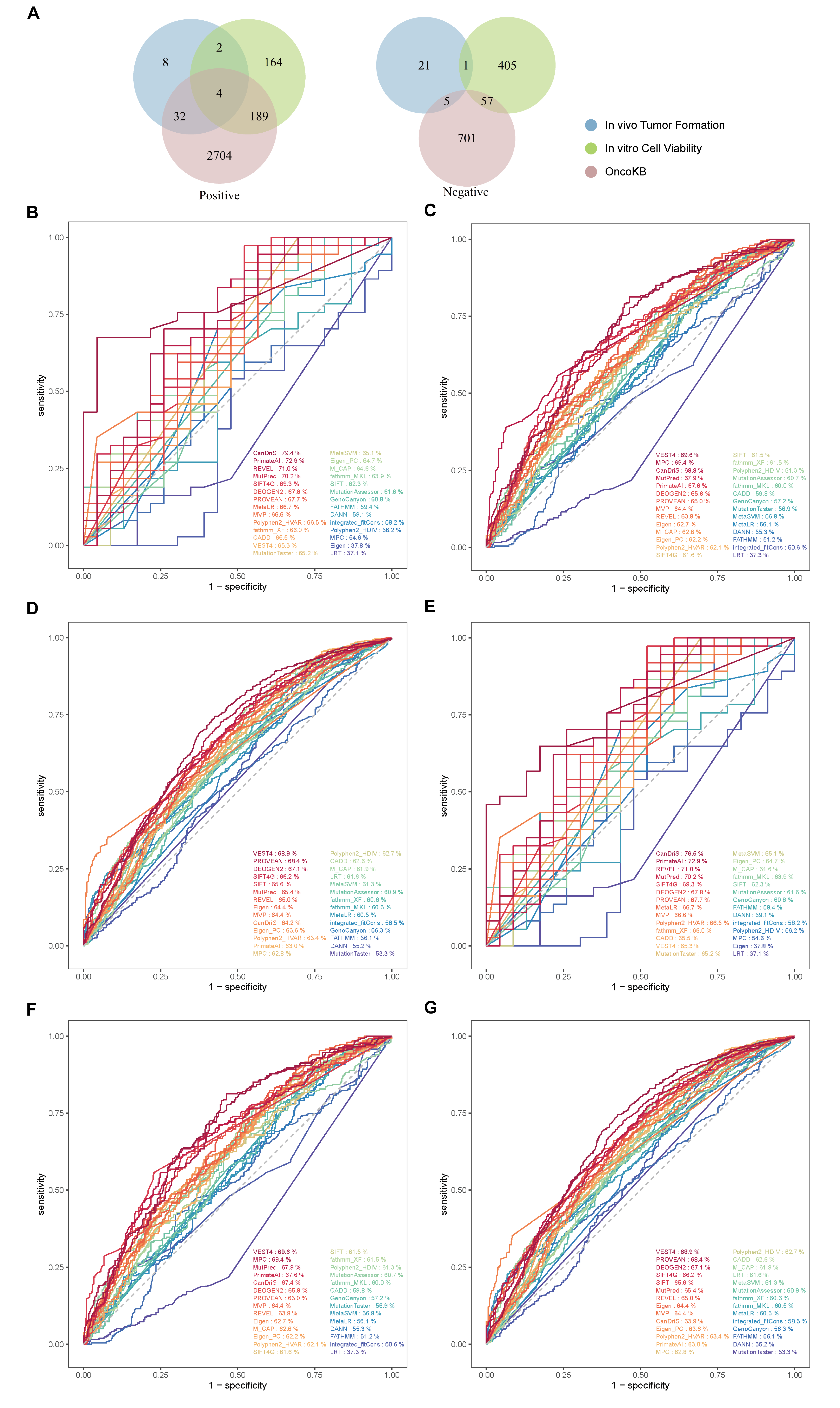


**Figure S9.** The performance of two-component mixture model based on the benchmark analysis. (A) Venn diagram of positive and negative samples in the three benchmark datasets. (B—D) The ROC curves of Poisson-NBD* mixture model and other 27 algorithms in three benchmark datasets. The Poisson-NBD* model showed consistently excellent performance. Especially in the *in vivo* dataset, the Poisson-NBD* model achieved the highest AUC score of 79.4%, which was well ahead of the second-ranked PrimateAI (AUC = 72.9%). (E—G) The ROC curves of Poisson-Poisson mixture model and other 27 algorithms in three benchmark datasets (*in vivo* tumor formation benchmark, *in vitro* cell viability benchmark and OncoKB annotation benchmark, respectively). As expected, the Poisson-NBD* model achieved higher AUC scores in all three benchmark datasets, especially in the *in vivo* dataset, which improved the AUC from 76.5% to 79.4%.


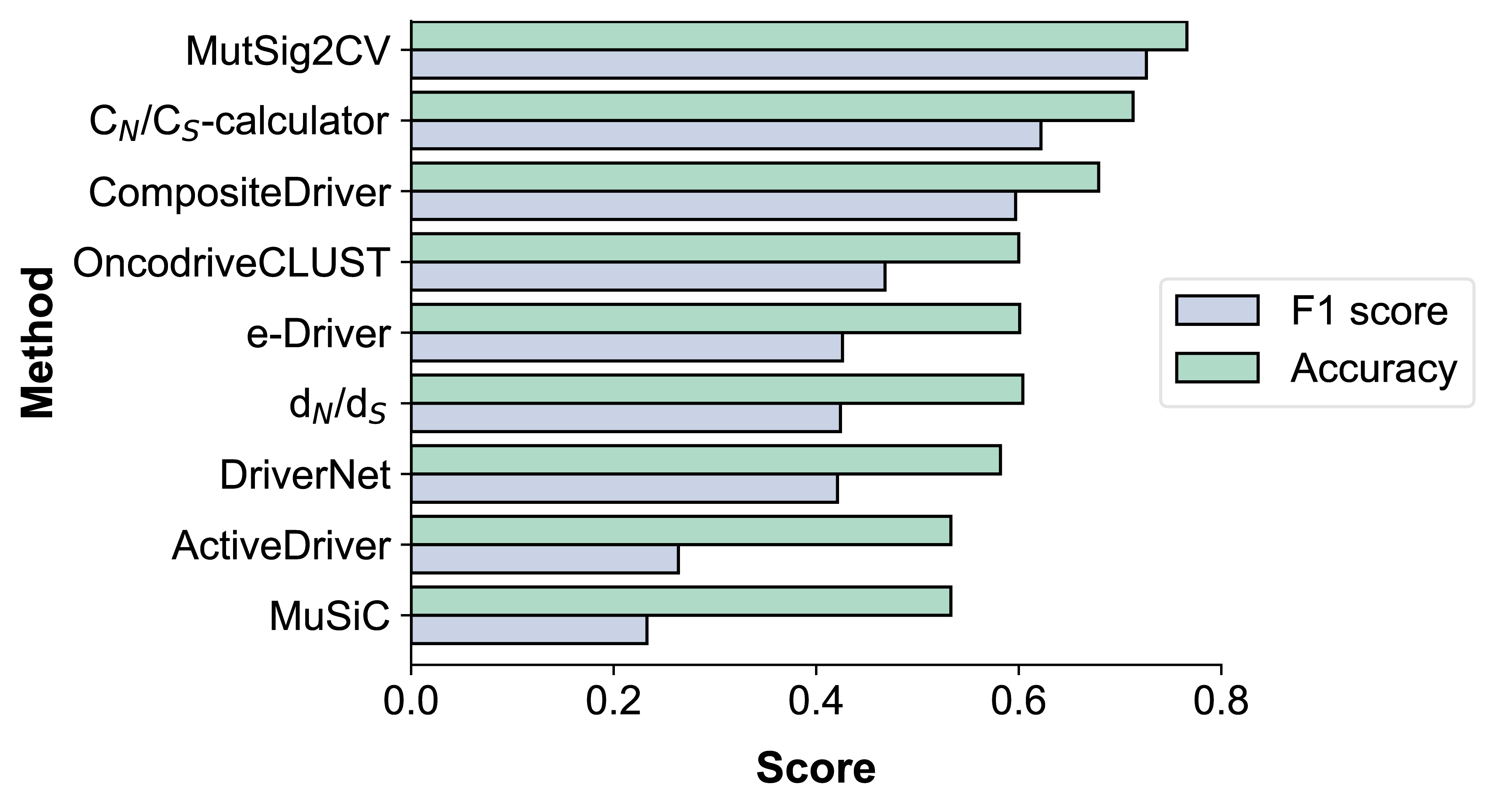


**Figure S10.** The F1 score and accuracy of all the driver genes predicted by *C_N_/C_S_*-calculator (without removal of six suspicious driver genes) and other eight driver gene prediction methods.

## Supplementary Tables

**Supplementary Table 1. Information of positively selected driver genes.**

**Supplementary Table 2. Information of weakly positively selected mini-driver genes.**

**Supplementary Table 3. Information of conserved mini-driver genes.**

**Supplementary Table 4. Suspicious driver gene list used for ‘Filter Dubious Genes’ option.**

**Supplementary Table 5. Manually collated literature evidence for novel driver genes.**

**Supplementary Table 6. Therapeutic implication of driver genes in the OncoTriMD database. Related to Figure 3C.**

**Supplementary Table 7. The ‘Likely Pathogenic’ or ‘Pathogenic’ mini-driver sites annotated by Clinvar database.**

**Supplementary Table 8. The driver genes with labeled level in OncoTriMD database.**

**Supplementary Table 9. The overlap between conserved mini-driver genes and human housekeeping genes.**

**Supplementary Table 10. Variant function prediction of the mini driver mutation *SMU1* (p.R346C).**
